# Transcriptomic Analysis Reveals Priming of The Host Antiviral Interferon Signaling Pathway by Bronchobini^®^ Resulting in Balanced Immune Response to Rhinovirus Infection in Mouse Lung Tissue Slices

**DOI:** 10.3390/ijms20092242

**Published:** 2019-05-07

**Authors:** Stella Marie Reamon-Buettner, Monika Niehof, Natalie Hirth, Olga Danov, Helena Obernolte, Armin Braun, Jürgen Warnecke, Katherina Sewald, Sabine Wronski

**Affiliations:** 1 Department of Preclinical Pharmacology and In Vitro Toxicology, Fraunhofer Institute for Toxicology and Experimental Medicine, Biomedical Research in Endstage and Obstructive Lung Disease Hannover (BREATH), Member of the German Center for Lung Research (DZL), Member of Fraunhofer international Consortium for Anti-Infective Research (iCAIR), 30625 Hannover, Germany; stella.reamon-buettner@item.fraunhofer.de (S.M.R.-B.); monika.niehof@item.fraunhofer.de (M.N.); olga.danov@item.fraunhofer.de (O.D.); helena.obernolte@item.fraunhofer.de (H.O.); armin.braun@item.fraunhofer.de (A.B.); katherina.sewald@item.fraunhofer.de (K.S.); 2 Heel GmbH, 76532 Baden-Baden, Germany; natalie.hirth@heel.de (N.H.); juergen.warnecke@heel.de (J.W.)

**Keywords:** rhinovirus, precision-cut lung slices, immunomodulation, interferon signaling pathway, transcriptomics, Bronchobini^®^

## Abstract

Rhinovirus (RV) is the predominant virus causing respiratory tract infections. Bronchobini^®^ is a low dose multi component, multi target preparation used to treat inflammatory respiratory diseases such as the common cold, described to ease severity of symptoms such as cough and viscous mucus production. The aim of the study was to assess the efficacy of Bronchobini^®^ in RV infection and to elucidate its mode of action. Therefore, Bronchobini^®^’s ingredients (BRO) were assessed in an ex vivo model of RV infection using mouse precision-cut lung slices, an organotypic tissue capable to reflect the host immune response to RV infection. Cytokine profiles were assessed using enzyme-linked immunosorbent assay (ELISA) and mesoscale discovery (MSD). Gene expression analysis was performed using Affymetrix microarrays and ingenuity pathway analysis. BRO treatment resulted in the significant suppression of RV-induced antiviral and pro-inflammatory cytokine release. Transcriptome analysis revealed a multifactorial mode of action of BRO, with a strong inhibition of the RV-induced pro-inflammatory and antiviral host response mediated by nuclear factor kappa B (NFkB) and interferon signaling pathways. Interestingly, this was due to priming of these pathways in the absence of virus. Overall, BRO exerted its beneficial anti-inflammatory effect by priming the antiviral host response resulting in a reduced inflammatory response to RV infection, thereby balancing an otherwise excessive inflammatory response.

## 1. Introduction

Every year millions of people are affected by respiratory tract infections causing the common cold. Symptoms include rhinorrhea, nasal congestions, sore throat, headache, sneezing, and coughing [1,2]. Despite this rather mild course of disease, and its usually self-limiting progression with recovery in approximately 7–10 days, the common cold continues to be one of the most frequent respiratory disease and has a high socioeconomic impact due to the high prevalence [3,4].

Human rhinovirus (RV) is the predominant pathogen causing the common cold [2,5,6]. RV is a non-enveloped positive single stranded RNA virus of the picornaviridae family with more than 150 known serotypes classified into groups A, B and the more recently discovered group C [7,8,9,10]. RV pathology in healthy humans has recently been reviewed in detail [11]. At the airway mucosal site, RV invades the epithelial cells as the primary target using different pathways for entry into the host cells [7,10,12]. Major group rhinoviruses comprising about 90% of RV serotypes A and all of group B use the human intercellular adhesion molecule 1 (ICAM1). Minor group rhinoviruses, i.e., the remaining serotypes of group A, use receptors of the low density lipoprotein receptor (LDLR) family [13,14,15]. For rhinovirus C, the receptor was recently discovered to be the cadherin-related family member 3 [16]. Upon uncoating and release of the viral RNA into the cytosol, replication is initiated leading ultimately to the release of new virions from the cell without inducing direct cytopathic effects. RV is able to replicate in epithelial cells of the upper and lower airways [17,18]. In contrast to major group RV using ICAM1, the LDLR-mediated infection with minor group serotypes, e.g., RV1b, has been described to be applicable in mouse models [19,20,21].

Once RV has invaded the epithelial cells, it is recognized by various pathogen associated pattern recognition receptors. The single-stranded RNA (ssRNA) as well as the double-stranded RNA (dsRNA) generated during replication are recognized by TLR3, TLR7 and TLR8 as well as RLR such as retinoic acid-inducible gene I (RIG-I, alias DExD/H-box helicase 58 (DDX58)) and melanoma differentiation-associated protein 5 (MDA-5, alias interferon induced with helicase C domain 1 (IFIH1)) [7,11,22,23,24,25,26,27,28,29]. These toll like receptors (TLRs) and RIG-I like receptors (RLRs) then activate interferon regulatory factors (IRFs), for instance, IRF7/IRF3 and induce secretion of type I and III interferons (IFN), which in turn, via autocrine/paracrine activation of type I IFN receptor activate the janus kinase (JAK)/ signal transducer and activator of transcription protein (STAT) signaling pathway [30]. This again leads to activation of a multitude of IFN inducible genes (ISGs) such as interferon-induced protein with tetratricopeptide repeats (IFIT) and 2′-5′-oligoadenylate synthetase 1 (OAS1) and eventually leads to expression of a multitude of IFN responsive genes. IFITs and OAS1 are involved in the inhibition of transcription of viral RNA, and IFITs have been described to also be important regulators of apoptosis [31]. IFNs themselves restrict viral replication and elicit antiviral resistance in adjacent cells. Induced chemokines, for example, C-X-C motif chemokine ligand 8 (CXCL8, alias interleukin 8 (IL8)), C-X-C motif chemokine ligand 10 (CXCL10, alias interferon gamma-induced protein 10 (IP10)), C-C motif chemokine ligand 5 (CCL5, alias regulated on activation, normal T cell expressed and secreted (RANTES)), and C-C motif chemokine ligand 2 (CCL2, alias monocyte chemoattractant protein 1 (MCP1)) recruit and activate immune cells, such as neutrophils, natural killer (NK) cells, and cytotoxic T cells. NK cells are an important early source of IFN-gamma (IFNG) [7]. Besides triggering the antiviral interferon response, recognition of virus via pathogen-associated molecular patterns (PAMPs)/ pattern recognition receptors (PRRs) (e.g., TLR2) independent of endocytosis and replication, stimulates a range of other signaling pathways including NFkB, not only in epithelial cells but also in macrophages [32,33,34]. Activation of NFkB pathway is pivotal in the regulation of inflammatory immune responses, e.g., the induction of inflammatory cytokines such as IL6, tumor necrosis factor α (TNF), and IL1β (IL1B) [35]. NFkB target genes include genes for pro-inflammatory cytokines (e.g., IL1, IL2, IL6, IL12, TNF), adhesion molecules (e.g., intercellular adhesion molecule, vascular cell adhesion molecule), chemokines (e.g., CXCL8), acute phase proteins, and inducible enzymes (e.g., nitric oxide synthase 2 (NOS2) and prostaglandin-endoperoxide synthase 2 (PTGS2), also known as cyclooxygenase 2 (COX2)) [35,36]. Many of the common cold symptoms have been linked to the release of these mediators, especially the pro-inflammatory cytokines IL1B and IL6 [37,38].

Owing to the vast number of different RV serotypes with its antigenic diversity, no vaccines are available until now. Despite some reports of anti-RV activity, e.g., for pleconaril, none of the existing antiviral drugs have been licensed as of yet for use in RV infection. Nonetheless, research with the most prominent candidates such as rupintrivir and novel analogs is underway (recently reviewed in [39]). Thus, current therapy is limited to symptomatic treatment. Natural medicines have long been neglected in the scientific community despite their long tradition in use to combat common colds, due to the sparse scientific evidence of their benefit as a complementary therapy for respiratory tract infections [40,41].

Bronchobini^®^ has been used for years on a daily basis in pediatric practice for treating inflammatory respiratory diseases. It consists of dilutions of plant extracts of *Atropa belladonna* (D5), *Bryonia* sp. (D3), *Cetraria islandica* (D3), *Drosera* sp. (D3), and *Psychotria ipecacuanha* (D3) and has been described to ease the severity of symptoms of common cold disease, hypothesized by modulating inflammatory processes. According to Commission D of the Federal Institute for Drugs and Medical Devices, *Bryonia* sp. and *Atropa belladonna* are indicated for the treatment of respiratory inflammation. *Cetraria islandica*, *Drosera* sp., and *Psychotria ipecacuanha* are used for the treatment of bronchitis, cough, and viscous mucus production.

Despite its known anti-inflammatory properties and long use based on its beneficial effects in reducing common cold symptoms, the mode of action of Bronchobini^®^, especially in modulating the antiviral immune response, has so far not been elucidated. Therefore, the present study aimed to investigate the efficacy and mode of action of Bronchobini^®^ ingredients (BRO) in an ex vivo RV infection in mouse precision-cut lung slices (mPCLS). PCLS as organotypic tissue contains all cell types present in the lung, which can be cultured ex vivo with a maintained tissue viability and response to external stimuli, closely resembling the lower respiratory tract immune response observed in humans in vivo [42]. Therefore, PCLS are a valuable tool to study respiratory infection and efficacy of pharmacological interventions. In recent years, we and others have established infection of PCLS ex vivo to study the pathomechanisms of respiratory tract infections [43,44,45,46,47,48].

Here we show scientific evidence of the anti-inflammatory effect of BRO during virus induced respiratory tract inflammation using the PCLS ex vivo rhinovirus infection model. PCLS as an immunocompetent tissue enabled analysis of BRO effects on both the antiviral and inflammatory immune response. Using in-depth whole genome expression and pathway analysis, we demonstrate that BRO’s beneficial action is not only based on its anti-inflammatory properties, but also its ability to specifically prime the antiviral immune response to invading virus. This leads to a balanced antiviral response, thereby preventing excess production of inflammatory mediators associated with symptoms and disease severity.

## 2. Results

### 2.1. BRO Reduced RV-Induced Release of Pro-Inflammatory and Antiviral Cytokines

**Figure 1:**
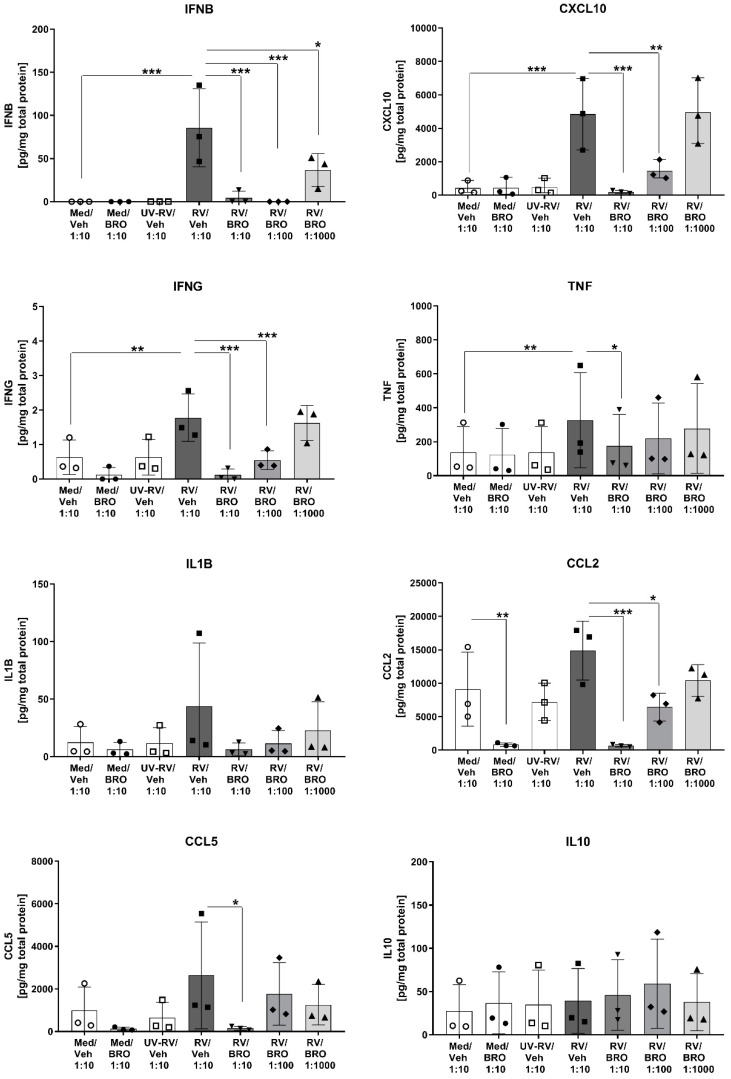
Bronchobini^®^’s ingredients (BRO) reduced rhinovirus- (RV) induced cytokine and chemokine release. Mouse precision-cut lung slices (PCLS) were infected with rhinovirus (RV) or sham-infected with medium (Med) or UV-inactivated, replication-deficient RV (UV-RV) in the presence of BRO (dilution 1:10, 1:100, 1:1000) or vehicle control (Veh, dilution 1:10). Cytokine protein levels were measured by ELISA or mesoscale discovery (MSD) in culture supernatants 24 h p.i. and normalized to the respective total protein content. Scatter plots with bars show mean + SD for *n* = 3 independent experiments with individual plots showing the mean of two biological replicates (duplicate wells with two PCLS each) per experiment. Each experiment was performed with PCLS pooled from three mice. *; **; *** indicate significance with *p* ≤ 0.05; 0.01; 0.001 according to a one-way ANOVA with Sidak’s multiple comparison post-hoc test.

An active RV infection was elicited ex vivo in the mouse lung slices and induced an antiviral host immune response. RV, but not the replication-deficient virus inactivated by ultraviolet (UV) light irradiation, induced the production and release of key cytokines in the antiviral host response such as Interferon β (IFNβ), chemokine (C-X-C) motif ligand 10 (CXCL10), and IFNG (Figure 1). This was not due to unspecific cytotoxic effects, as no increase in lactate dehydrogenase (LDH) release was observed (Figure S1), and tissue viability was maintained throughout the experiment. Furthermore, BRO had no cytotoxic effect as treatment in all concentrations did not impair tissue viability (Figure S1).

Treatment with BRO had a dose-dependent impact on this RV-induced antiviral cytokine release, with a significant reduction of IFN-beta (IFNβ), CXCL10, and IFNG to baseline levels in the BRO high dose group (Figure 1). IFN-alpha (IFNα) was only detectable in the RV and RV/BRO low dose group, implicating a slight induction by RV and a suppression by BRO high and medium dose, however, levels were below the valid detection range (data not shown). The pro-inflammatory cytokine tumor necrosis factor (TNF) was significantly induced by RV, while the increase in interleukin 1 β (IL1β) was only marginal and did not reach statistical significance. Interleukin 6 (IL6) and keratinocyte chemoattractant (KC), the neutrophil-attracting chemokine IL8 equivalent in the mouse, were elevated above the detection limit already in the baseline condition (data not shown). Thus, both cytokines were induced in the culture independent of RV infection. BRO treatment seemed to suppress IL6 release, as in the high dose group IL6 levels were detectable within the valid detection range of the assay (data not shown). Chemokines CCL5 (RANTES) and CCL2 (MCP1), biomarkers for a later induced inflammatory cell infiltration, showed elevated levels upon RV infection but did not reach statistical significance. Both chemokines were, however, significantly inhibited by BRO in the high dose group, and CCL2 was still significantly suppressed in the medium dose group (Figure 1). Additionally, BRO even suppressed the baseline levels of CCL2 present in the PCLS. For the anti-inflammatory cytokine IL10 no significant changes were observed between all groups (Figure 1). All BRO effects were compared to the respective vehicle control (0.92% ethanol) and therefore confirmed that the observed effects were specifically induced by the active ingredients. The observed effect of BRO suppression of the immune response seemed not to be due to a direct antiviral activity of BRO, as comparable amounts of virus load were detected in all samples infected with RV with or without treatment (Figures S2 and S3).

### 2.2. Analysis of BRO Mode-of-Action Using Whole Transcriptome Analysis

We carried out a whole transcriptome analysis to gain insights into the mode of action of Bronchobini^®^. Therefore, a genome-wide analysis of differentially expressed genes (DEGs) was performed to assess the gene expression induced by RV infection of the mPCLS ex vivo and to analyze the modulation of gene expression by BRO under both baseline (uninfected) condition and during RV infection (Table 1). The results show that treatment with BRO at 1:10 dilution (high dose) resulted in the induction of a multitude of significant DEGs, both under baseline condition (uninfected) and upon RV infection (6693 DEGs and 5665 DEGs, respectively). The RV infection itself induced a set of 692 DEGs. In the RV infection situation, BRO showed a clear dose-dependent response in DEGs. The vehicle for BRO treatment did not induce this regulation, confirming the specificity of the effects due to the active ingredients. On the basis of these results, further analysis was focused on the BRO 1:10 diluted high dose group, further referred to as BRO, showing the most robust regulation.

**Figure 2:**
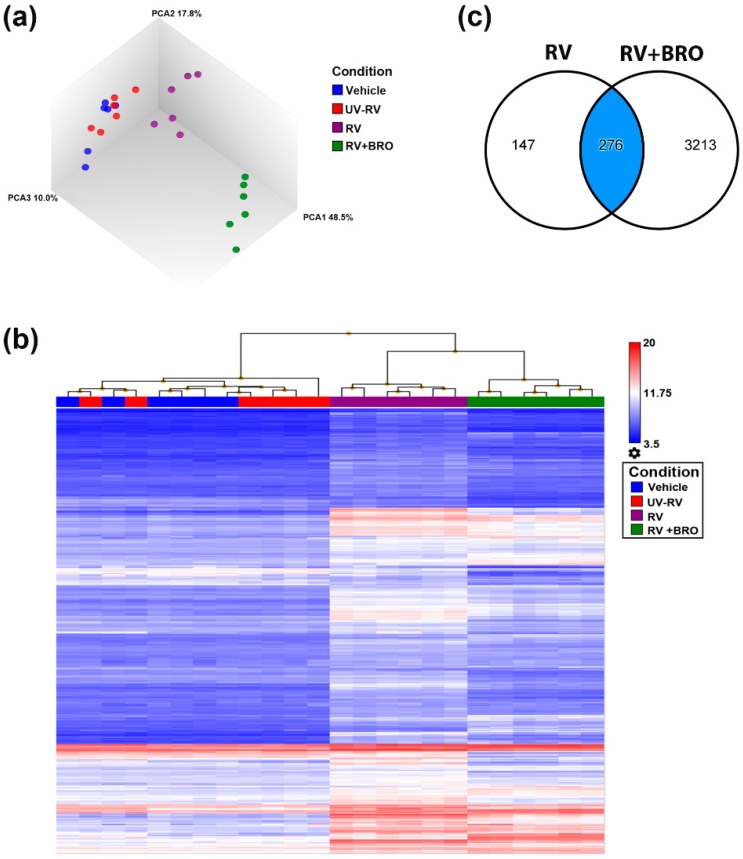
Bioinformatic analyses of whole transcriptome datasets. (**a**) Principal component analysis (PCA) of transcriptome datasets obtained from mPCLS infected with rhinovirus (RV) or sham-infected with medium or UV-inactivated RV (UV-RV) in the presence of Bronchobini^®^’s ingredients (BRO) or vehicle control. (**b**) Heat map of unsupervised hierarchical clustering based on DEGs obtained in RV (both generated using Transcriptome Analysis Console Software TAC 4.0). (**c**) Venn diagram showing overlap of DEGs by RV and RV+BRO (generated using an ingenuity pathway analysis (IPA) using mapped genes and filter criteria of DEGs at fold change <−2 or >2; ANOVA *p*-value < 0.05). Vehicle = sham-infected with medium and treated with vehicle, UV-RV = sham-infected with UV-inactivated RV and treated with vehicle, RV = infected with RV and treated with vehicle, RV + BRO = infected with RV and treated with BRO (1:10 dilution).

We employed various bioinformatic tools to characterize the transcriptome datasets, and delineate the biological meaning of the transcriptional activity modulated by BRO treatment during RV infection. The principal component analysis (PCA) shows the robustness of the datasets and a clear distinct grouping of baseline samples (vehicle and UV-inactivated RV), RV infection, and RV-infected and BRO treated samples (Figure 2a). The response is shown in more detail in the heat map of the unsupervised hierarchical clustering, where RV response displayed a distinct gene expression pattern compared to medium and UV-RV infected samples, and BRO treatment again showed a distinct expression profile (Figure 2b). Comparison analysis revealed that approximately 2/3 of the RV induced DEGs were significantly regulated by BRO (276 common regulated genes, Figure 2c). The individual unique or common DEGs are listed in Tables S1–S3.

Among the RV and BRO mutual DEGs, the most strongly RV induced genes were related to the interferon induced antiviral immune response, such as those genes encoding for interferon-inducible proteins *Ifit2* and *Ifit3*, *Cxcl10*, *Irf7*, *Mx1* and *Oas1/2*. These, in turn, were significantly downregulated in the presence of BRO (all fold changes (FC) listed in Table S1). Accordingly, detailed bioinformatic analyses using an ingenuity pathway analysis (IPA) showed the interferon signaling pathway to be the most prominent of the top regulated canonical pathways induced by RV infection but inhibited in the presence of BRO (see Figure S4 showing the top-scoring canonical pathways).

#### 2.2.1. BRO Modulation of RV-Induced Interferon Signaling Pathway

Based on its strong regulation and its crucial role in the antiviral host response, we evaluated the virus-induced interferon response in more detail to delineate the mode of action of Bronchobini^®^. IPA analysis predicted IRFs as well as interferons themselves to be main upstream regulators of the gene expression changes observed, and these were activated by RV infection, but inhibited in the presence of BRO (Figure S5). However, as the production and release of the interferons already were observed to be less induced in the presence of BRO (both observed in gene expression as well as on the protein level), the subsequent inhibition of the interferon induced signaling pathway and respective regulation of interferon target genes was only a secondary effect.

**Figure 3:**
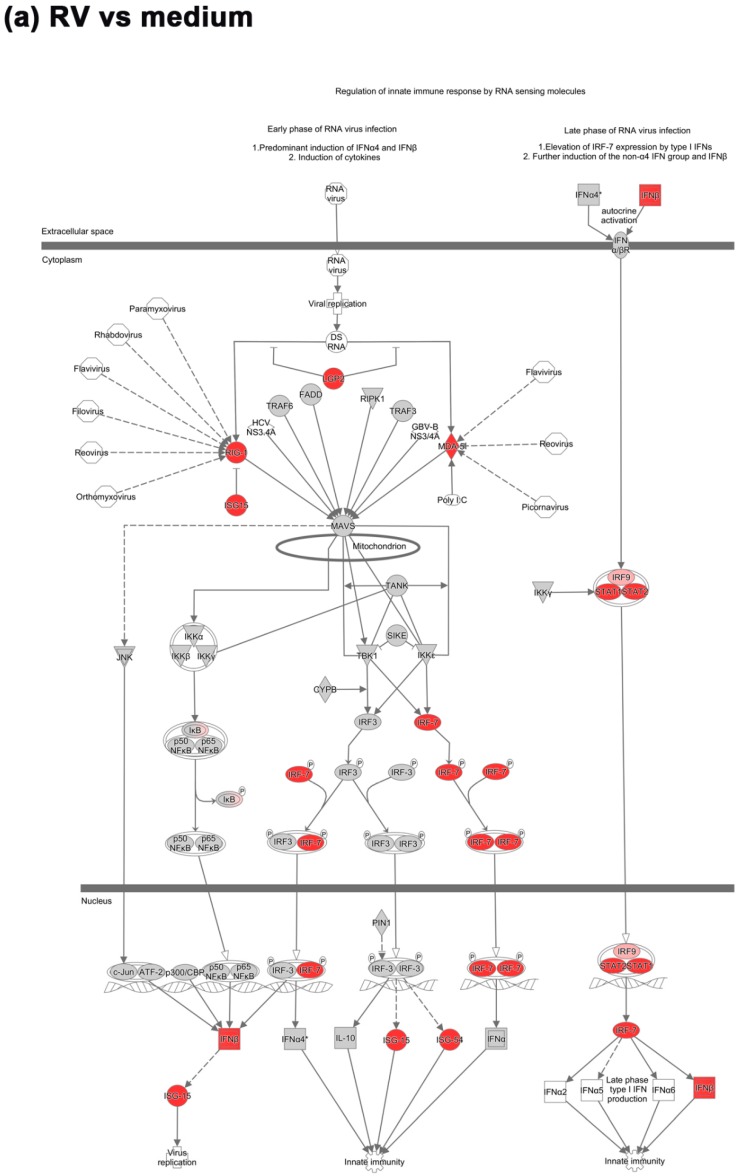

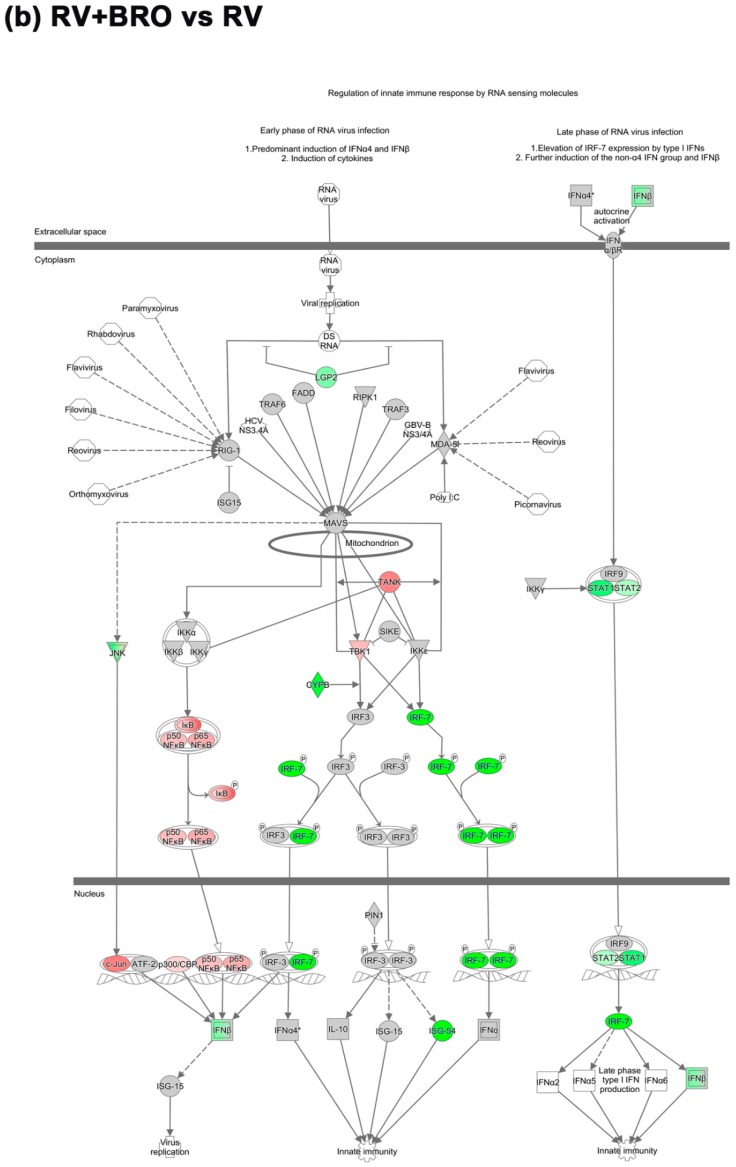
BRO modulated virus-induced activation of the interferon response. (**a**) Regulation of interferon response factor (IRF) activation by PRR (pattern recognition receptors) during RV infection and (**b**) BRO effect on RV induced IRF activation by PRR. Pathway analyzed using IPA. Red or pink (upregulated), green (downregulated), gray or white (does not meet cut-off criteria or not involved in the pathway). Black solid arrows (direct interaction); black dotted arrows (indirect interaction).

We, therefore, further analyzed the upstream regulatory pathway involved in the recognition of virus and induction of the early interferon response and how they were modulated by BRO during RV infection. Key molecules mediating the recognition of virus and induction of the antiviral interferon response were upregulated under RV infection, while in contrast were downregulated in the presence of BRO (Figure 3).

The initial recognition of replicating virus seemed not to be suppressed by BRO, as the RV induced upregulation of the RLR encoding genes in the mouse PCLS e.g., *Rig-1* (*Ddx58*) and *Mda-5* (*Ifih1* (FCs 10.4 and 22.5 vs. uninfected control, respectively) was not significantly altered in the presence of BRO (Figure 3). However, the gene expression of Rig-I-like receptor gene *Lgp2* (*Dhx58*), a positive regulator of RIG-I and MDA-5 signaling, which was also upregulated in RV infection (FC 17.9 vs. uninfected control) was significantly inhibited in the presence of BRO (FC −3.7 vs. RV; Figure 3 and Table S4).

The virus-induced RIG-I/MDA-5 mediated activation of IRFs was strongly modulated by BRO. RV induced strong upregulation predominantly of interferon regulatory factor encoding genes such as *Irf7* (FC 53.5) and *Irf9* (FC 3.9). In the presence of BRO, *Irf7* was strongly downregulated (FC −9.4 vs. RV), while *Irf9* was not significantly modulated (Figure 3 and Table S4). *Irf3* as the main interaction partner of *Irf7* was not significantly upregulated by RV or modulated in the presence of BRO on the transcriptional level.

In line with the observed IRF regulation, the expression of type I and III interferon genes, i.e., *Ifnb1* and interferon lambda 3 (*Ifnl3*) that were significantly induced by RV infection (FC 9.3 and 2.9, respectively) were reduced, but not completely shut down to the baseline level, in the presence of BRO (FC −3.7 and −2.6 vs. RV, respectively; Figure 3, Table S4, and data not shown). *Ifna* was not found to be significantly regulated in both conditions.

Interferons, in turn, activate the JAK/STAT signaling pathway in response to infection. Accordingly, we observed a strong upregulation of the transcription regulator genes *Stat1* and *Stat2* (FC 33.6 and 13.9, respectively; Figure 3a). In the presence of BRO, both were significantly downregulated (FC −5.8 and −2.5, respectively) compared to untreated RV infection (Figure 3b). In line with this, IFN-driven JAK/STAT signaling upon RV infection led to the expression of a multitude of interferon stimulated genes (ISGs) such as IFITs. *Ifit2* (*Isg54*) was strongly upregulated by RV (FC 149.6), and downregulated in the presence of BRO (FC −8.6) as compared to RV alone (Figure 3 and Table S4). *Ifit3* was highly expressed in RV infection (FC 123.0) but reduced (FC −6.3) in the presence of BRO as compared to untreated RV infection (data not shown). *Isg15* was upregulated by RV (FC 10.3), but not significantly altered in the presence of BRO. *Ifit1* (*Isg56*) was upregulated by RV (FC 10.0) and even further induced in the presence of BRO (FC 3.0; data not shown). Another example of ISGs that were regulated by BRO was *Oas1*, which was strongly induced by RV (FC 37.2), while significantly downregulated with BRO (FC −9.0; data not shown). All DEGs with fold changes and p-values are given in Tables S1–S4.

Importantly, while BRO treatment down-regulated the RV induced activation of this pathway and hence the interferon response, this was not completely shut-down but still activated compared to uninfected medium control (see Figure S6 and Table S4).

#### 2.2.2. BRO Modulation of NFkB-Mediated Pro-Inflammatory Response to Rhinovirus

Besides the modulation of the antiviral interferon response, a strong modulation of the pro-inflammatory response by BRO was observed (data not shown, fold changes see Tables S1–S3). In line with the observed increase in pro-inflammatory cytokine protein levels, RV induced significant induction of the gene expression of *Tnf* (FC 4.1) and *Il6* (FC 15.6). *Il1b* gene expression was not significantly upregulated upon RV infection, however, in the presence of BRO it was downregulated compared to the uninfected controls (FC −2.5). Furthermore, caspase 1 (*Casp1*), which cleaves IL1β precursor to its mature form, was significantly upregulated in RV infection (FC 3.0) but in the presence of BRO *Casp1* gene expression was completely shut down (FC −2.8). The RV-induced upregulation of *Il6* gene expression (FC 15.6) was not altered in the presence of BRO, but as described above Il6 protein levels were strongly reduced in presence of BRO (Figure 1). Interestingly the receptor (*Il6r*) gene expression, which was not differentially regulated upon RV infection itself, was altered in the presence of BRO. *Il6r* and *Il6st* (gp130) were upregulated (FC 4.3 and 3.0 vs. RV alone).

**Figure 4:**
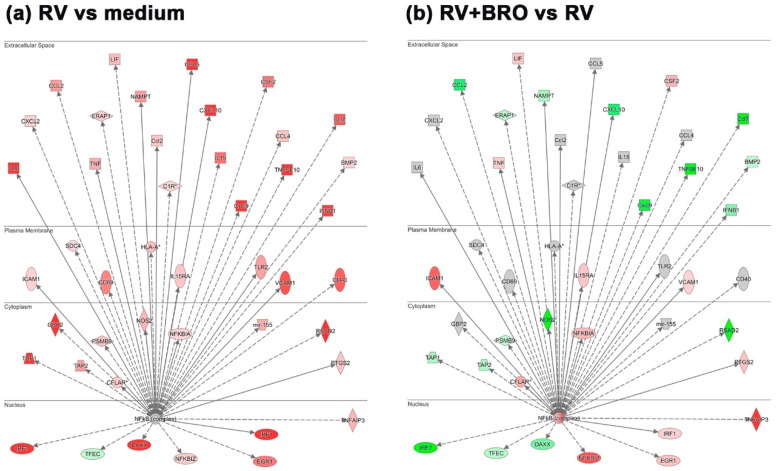
BRO modulation of RV-induced NFkB target gene expression. (**a**) Regulation of NFkB target gene expression during RV infection and (**b**) BRO effect on RV induced NFkB target gene expression. Analysis obtained using IPA. Red or pink (upregulated), green (downregulated), gray or white (does not meet cut-off criteria or not involved in the pathway). Black solid arrows (direct interaction); black dotted arrows (indirect interaction).

IL1B and IL6 as predominantly symptom-associated cytokines, as well as a multitude of other pro-inflammatory mediators, are known to be induced by RV via the NFkB pathway. We thus further investigated how the expression of genes participating in this pathway was regulated in the PCLS ex vivo infection and how it was modulated by BRO. Upstream analysis for NFkB as a transcriptional regulator showed that genes encoding for pro-inflammatory chemokines and cytokines as well as interferon response regulators were induced by RV via this pathway. A similar induction of RV was also observed for genes linked to leucocyte infiltration (*Icam1*, *Vcam1*) and activation (*Cd40*, *Cd69*), as well as inflammation associated enzymes such as *Nos2* and molecules involved in prostaglandin synthesis (*Ptgs2*; Figure 4a and Table 2). BRO treatment resulted in a significant differential modulation of these genes (Figure 4b and Table 2). Further molecules known to be involved in these biological processes, were also regulated by RV and modulated by BRO, such as *Icos*/*Icosl* as T-cell costimulatory molecules, as well as *Ptges* and *Nampt* associated with prostaglandin production (Table 2). Additionally, further genes associated with apoptosis induction (*Tnfsf10* also known as *Trail*) as well as apoptosis inhibition (*Tnfaip3*) were upregulated by RV (Figure 4a).

While an overall downregulation of RV-induced inflammatory chemokines and cytokines was observed in the presence of BRO, as already described above, genes associated with leukocyte infiltration, such as *Icam1* and *Vcam1* were upregulated (Figure 4b and Table 2). Leukocyte activation markers *Cd40* and *Cd69* were not significantly altered, while *Icos*/*Icosl* were further upregulated (Table 2). Furthermore, RV-induced expression of *Nos2* was observed to be reduced in the presence of BRO, and while *Ptgs2* expression was further enhanced, *Ptges* and *Nampt* were downregulated in the presence of BRO indicating a reduction of prostaglandin E_2_ (PGE_2_) synthesis (Figure 4b and Table 2). Additionally, NFkB regulated signaling of apoptosis was modulated by BRO, as e.g., the RV-induced expression of *Tnfsf10* was strongly downregulated in the presence of BRO, while the genes encoding for apoptosis inhibitory factors *Tnfaip3* and *Traf1/2* were upregulated by BRO as compared to RV infection alone (Figure 4b and Table 2).

Overall, BRO treatment resulted in a reduced but not abolished antiviral and pro-inflammatory immune response to the rhinovirus infection by downregulating RV-induced signaling pathways.

#### 2.2.3. BRO Primes Antiviral and Pro-Inflammatory Host Signaling Pathways in Absence of Virus

**Figure 5:**
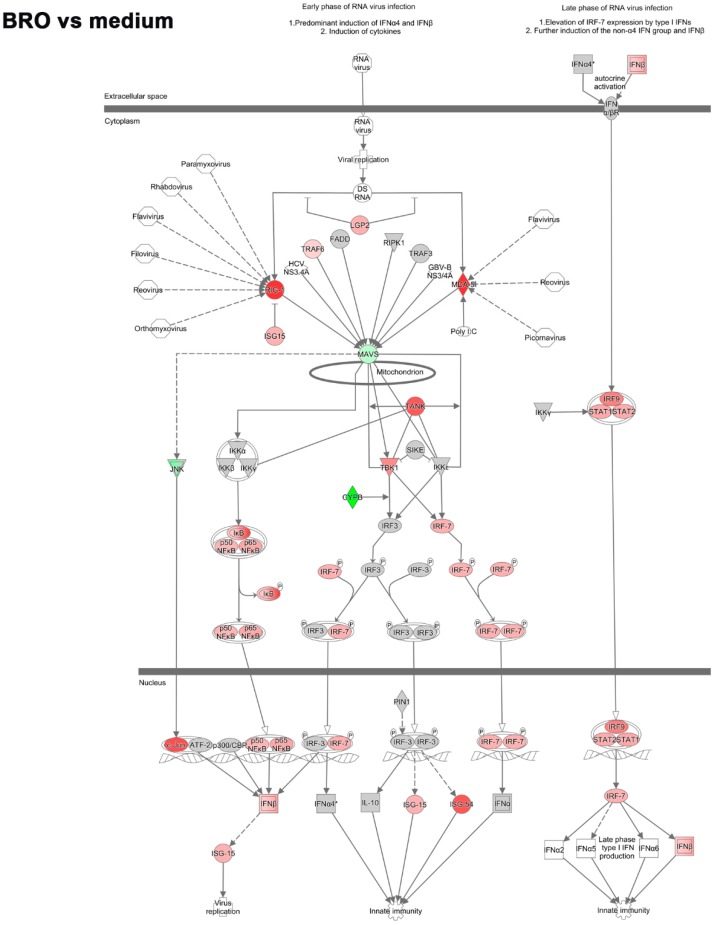
BRO priming of interferon response pathway in the absence of virus. Regulation of the interferon response factor (IRF) activation by PRR (pattern recognition receptors) by BRO in the absence of virus. Pathway obtained using IPA. Red or pink (upregulated), green (downregulated), gray or white (does not meet cut-off criteria or not involved in the pathway). Black solid arrows (direct interaction); black dotted arrows (indirect interaction).

**Figure 6:**
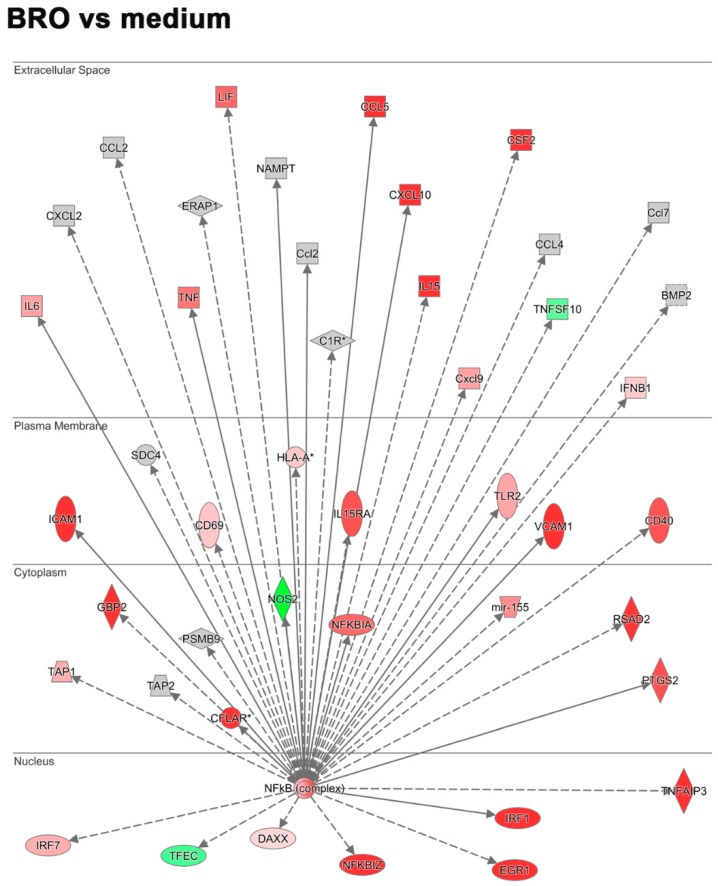
BRO priming of NFkB target gene expression in the absence of virus. Regulation of NFkB target gene expression by BRO in absence of virus. Analysis obtained using IPA. Red or pink (upregulated), green (downregulated), gray or white (does not meet cut-off criteria or not involved in the pathway). Black solid arrows (direct interaction); black dotted arrows (indirect interaction).

The above detailed reduction of the antiviral and pro-inflammatory host response to virus in the presence of BRO interestingly was not due to a suppression of these pathways by BRO in general, as in the uninfected condition BRO unexpectedly stimulated activation of these pathways leading to induction of IRF activating pathway (Figure 5) as well as NFkB pathway (Figure 6).

For example, the virus sensing RLRs *Rig-I* (*Ddx58*) and *Mda-5* (*IFIH1*) as well as *Lgp2* (*DHX58*) were upregulated in response to BRO treatment despite no virus was present (FC 10.2, 9.8, and 3.9, respectively). Accordingly, downstream signal molecules such as *Irf7* and *Irf9* were upregulated (FC 4.0 and 6.3). In line with this, *Ifnb1* was upregulated (FC 2.6). Furthermore, *Stat1/2* were upregulated (FC 4.3/4.1) as well as *Ifit2* (FC 8.2), and *Isg15* (FC 3.8; Figure 5 and Table S5).

Similarly, *NFkB* target gene expression was upregulated by BRO in the absence of virus. Gene expression of pro-inflammatory cytokines *Tnf* and *Il6* (FC 6.8 and 4.6, respectively) and chemokines *Ccl5* and *Cxcl10* (FC 15.3 and 18.9) was significantly upregulated by BRO. Interestingly, also the expression of the inhibitory IL6 receptor α gene (*Il6ra*) was upregulated (FC 4.3). Furthermore, NK cell activating cytokine gene *Il15* was upregulated (FC 12.6; Figure 6, Table S6). In the absence of virus, BRO also strongly upregulated gene expression of *Icam1* and *Vcam1* (FC 19.8 and 16.1) as well as costimulatory molecules such as *Cd40*, *Cd69* and *Icos/Icosl* (FC 8.2, 2.8 and 3.6/14.3, respectively). The inducible enzyme *Nos2* gene was downregulated (FC −8.0). *Ptgs2* was upregulated (FC 8.4), but *Ptges* downregulated (FC −5.5) by BRO, indicating no activation of prostaglandin E synthesis. Regarding modulation of apoptosis, *Tnfsf10* (*Trail*) was downregulated (FC −4.3) while inhibitory *Tnfaip3*, *Traf1*, and *Traf2* were strongly upregulated (FC 91.3, 11.6, and 8.1, respectively).

In summary, our data show that BRO per se activates host defense pathways, putting the host on alert, which seems to result in an improved, balanced host response to an invading virus while avoiding detrimental excess inflammation. Thus, Bronchobini^®^ could exert its observed beneficial effects in reducing disease symptoms by balancing the host immune response via its multifactorial modulation of virus-induced interferon and NFkB -mediated signaling.

## 3. Discussion

In the current study, we aimed to shed light into the mode-of-action of Bronchobini^®^ as a widely used pediatric therapy for the treatment of inflammatory respiratory diseases, e.g., the common cold. Therefore, we used an ex vivo model of respiratory tract RV infection using mouse precision-cut lung slices (PCLS) as viable, immunocompetent tissue with maintained microanatomy.

To achieve active infection in the mouse PCLS, rhinovirus minor group serotype 1b was used, which has been confirmed to actively infect mice as it binds to the cross-species conserved LDL receptor [21]; and can be used to study RV infection in mice [19,20,49].

We confirmed that by using RV1b, an active infection was induced in the mouse PCLS ex vivo and elicited a robust antiviral and pro-inflammatory response, as assessed by the release of a multitude of chemokines and cytokines. A certain variability in the cytokine response could be due to donor variances but was primarily because of variations in airway size and different proportions of cell subpopulations in PCLS as a complex organotypic tissue. Since circulation in PCLS ex vivo is absent, infiltration of cells cannot be mimicked. Nonetheless, as all lung cell types are present in PCLS within the intact lung microenvironment, the initiation of infection and induction of the innate immune response, i.e., early events in the pathogenesis, can be reflected. More important, the mediators released by the resident cells are valuable biomarkers for the later steps of the inflammatory host response. Cytokine response in PCLS stimulated with LPS has been shown to correlate to the in vivo response in humans [42]. One limitation in the current study in the interpretation of cytokine release is that for unspecific pro-inflammatory cytokines IL6 and KC, we observed already high protein levels in non-infected, untreated controls. However, despite this unspecific release of IL6 and KC, this was not the case for other mediators such as interferons and chemokines, which were specifically induced only upon RV infection. Applicability of PCLS as an ex vivo tissue mounting specific responses to diverse stimuli relevant to the in vivo situation and its value to study pharmacological intervention has been shown in numerous studies [42,45,46,50].

As expected, the ex vivo RV infection predominantly induced an antiviral host response via type I interferons, therefore in the PCLS ex vivo infection the response known to typically occur in the early stages of infection [51]. Antiviral type I interferons are produced by a variety of cells including airway epithelial cells, monocytes, and plasmacytoid dendritic cells (pDCs). PCLS contain all lung-resident cell types, including monocytes, macrophages, and dendritic cells, as well as resident NK and T cells [47], while the later influx of e.g., cytotoxic T cells during the adaptive response cannot be modeled in this ex vivo tissue. However, our results confirm that early mediators of the antiviral and pro-inflammatory host response are robustly stimulated in PCLS ex vivo and allow investigation of BRO effects on the early events of RV infection. Mediators such as CXCL10, IL1, and IL6 have been associated with symptoms and disease severity [37,52]. Despite its important function as a key mediator of the antiviral host response, CXCL10 has been discussed to also contribute to lung inflammation and that it is essential to limit excess CXCL10 production [53]. BRO reduced but did not abolish the RV-induced CXCL10 in our study, which indicates that it balances excess inflammation while maintaining the host’s ability to mount the necessary antiviral interferon response.

IFNs are induced by recognition of virus, thus less virus would induce less IFN secretion. The viral load measured by tissue culture infective dose 50 (TCID_50_) assay in homogenized PCLS was not affected by BRO treatment. However, airway epithelial cells as target cells for RV infection are only a small proportion compared to the whole PCLS. Furthermore, previous experiments have shown that only a small proportion of airway epithelial cells gets infected. Therefore, the amount of actually infected cells and release of new virions upon replication is low, which is reflected in the low amount of virus detected in the PCLS homogenate compared to the inoculum. The cytokine response of CXCL10, however, confirms an active, productive infection, especially as the replication-deficient UV-RV did not elicit CXCL10. No change of virus titer was observed upon BRO treatment, which was further confirmed by virus PCR quantification as a more sensitive method. Therefore, the reduced antiviral host response upon BRO treatment was not due to lower virus levels.

Importantly, abolishing the host response while the virus is still present would be detrimental, as no protective immune response could be mounted by the host leading to an uncontrolled virus spread. For patients with chronic lung diseases such as asthma evidence accumulates that an impaired antiviral interferon response to rhinovirus [54] seems to be responsible for prolonged and more severe infections leading to severe exacerbations of the disease. Studies have reported that the impaired antiviral response is not due to a deficient expression of the virus sensing pattern recognition receptors TLR3 and MDA5 but impaired signaling [54]. Interestingly, we observed that *Tlr3* was upregulated by RV infection, but was downregulated by BRO in mouse PCLS. While TLR3 usually mediates sensing of viral dsRNA during replication and leads to apoptosis of these virus infected cells, a recent study reported that rhinovirus suppresses the TLR3 mediated apoptosis induction by its viral 3C protease activity [55]. Thus, instead of a controlled removal of infected cells via apoptosis, an alternative necrotic death pathway leading to membrane rupture is induced, which could promote dissemination of new virions. By preventing TLR3 upregulation BRO therefore might prevent rhinovirus to induce and hijack this pathway.

Interestingly, we observed that BRO modulated the expression of several apoptosis-associated genes. For example, BRO downregulated the gene encoding TNF-related apoptosis-inducing ligand (TRAIL), while upregulating the gene expression of inhibitory factors such as tumor necrosis factor alpha induced protein 3 (TNFAIP3) and TNF receptor associated factor TRAF1/2. TRAIL has been shown to be pro-inflammatory in a RV1b mouse model of infection, promoting RV-induced airway hyperreactivity and inflammation, while knock out mice were protected from RV-induce pathology [56]. TNFAIP3 in turn has been shown to be critical in limiting inflammation by terminating TNF-induced NFkB responses and is a key molecule mediating tissue homoeostasis [57,58]. Thus, BRO might prevent excessive inflammation also by upregulation of TNFAIP3.

Limiting the pro-inflammatory immune response is essential, as host effector cells while combating the infection also contribute to tissue damage. The response therefore needs to be tightly regulated to prevent excessive inflammation and damage in the wake of the host defense. Our data revealed that BRO reduced but did not completely abolish the RV-induced activation of the antiviral interferon pathways, which indicates that it balances an otherwise potentially harmful excessive inflammatory response. For instance, BRO exhibited prominent regulatory effects on the virus induced activation of IRFs by pattern recognition receptors sensing virus. A dysfunction of IRF3/7 as main mediators of the type I and III interferon response have been described to be associated with increased morbidity and mortality in influenza infected mice [59]. Gene expression of *Irf7* was downregulated, but not completely suppressed by BRO, while that of *Irf3* was not regulated, indicating that BRO does not lead to a dysfunction of IRF3/7, but rather balances excessive activation. Interestingly, IRFs were not in general suppressed by BRO, as for example, *Irf5*, which was not induced by RV was upregulated by BRO treatment. As the human IRF5 has been associated with promoting macrophage activity and polarization to T helper type 1 (Th1)/Th17 response [60], this indicates that BRO positively supports the innate immune response to clear the viral infection. This is also in line with the observation, that while BRO suppressed inflammatory mediators, the activation of immune cell costimulatory molecules and maturation markers, e.g., CD40, CD69 was not suppressed or even enhanced (e.g. inducible T cell costimulatory (ICOS) and ICOS ligand (ICOSL)). Furthermore, even in the absence of virus BRO itself upregulated the expression of those genes indicating that it might exert a ‘priming’ function, alerting the immune response, which in turn might support to deal with the infection more efficiently. However, we only investigated this on a transcriptional level and future studies are needed to assess in detail if and how BRO activates specific immune cells as well as to test its efficacy in terms of e.g., killing efficacy of infected cells. As BRO is a multi-target, multi-component medication, the exact mode-of-action in terms of receptor interactions and target cells is unknown. Due to its multi-component composition, BRO presumably acts on multiple cell types including airway epithelial cells generating the interferon response to virus, as well as immune cells such as phagocytes.

One prominent effect of BRO was its ability to downregulate the NFkB-mediated pro-inflammatory host response, which could account for its beneficial effect to alleviate symptoms as observed in clinical use. IL1B is major pro-inflammatory, pyogenic mediator, which has been related to symptoms in RV infection [38]. Recognition of viral dsRNA is known to induce NOS2, PTGS2 (COX2) and IL1B in macrophages [61]. IL1B in turn further induces PTGS2 and PGES2 and thereby promotes prostaglandin synthesis.

We have observed that in presence of BRO, the RV induced production and release of these symptom-associated inflammatory mediators was reduced. BRO treatment led to a suppression of both *Il1b* as well as *Casp1* gene expression. Furthermore, enzymes in prostaglandin synthesis were differentially modulated indicating a reduced production of prostaglandin E, as a mediator of pain. BRO induced *Ptgs2* gene expression, while downregulating *Ptges*. This could indicate a shift in prostaglandin synthesis, as the PGE_2_ precursor prostaglandin H_2_ (PGH_2_) synthesized by PTGS2 might then preferably generate other prostaglandins, e.g., PGD2, which can act contrary to PGE_2_. Interestingly, a recent study reported that prostaglandin D suppressed virus-induced inflammasome activation, indicating its role in preventing excessive inflammation [62]. Furthermore, nicotinamide phosphoribosyltransferase (NAMPT), a metabolic enzyme known to also promote B cell maturation and inhibit neutrophil apoptosis [63], has been reported to play a role in PGE_2_ synthesis [64]. *Nampt* was upregulated by RV but reduced by BRO in the current study, further supporting the role of BRO in modulating PGE_2_ synthesis. However, to delineate the role of BRO in modulation of prostaglandin and potentially other eicosanoid synthesis, future studies need to measure eicosanoid levels upon RV infection and to assess the efficacy of BRO in reducing these mediators.

Beside IL1B, also IL6 is a major pyogenic cytokine, associated with disease symptom severity [38]. Surprisingly, we observed that BRO seemed to reduce IL6 protein levels, while *Il6* gene expression was not affected. However, post-transcriptional modulation of IL6 is present at different levels, as a strict regulation of IL6 is essential due to the fact that IL6 acts as an alarm signal [65]. Posttranscriptional regulation of IL6 expression is mediated as reviewed in detail by Tanaka et al. [65]. For example, mitogen-activated protein kinase (MAPK) p38α promotes stabilization of IL6 mRNA, and this was downregulated in the presence of BRO. Furthermore, regulatory RNase1 (regnase1), also known as ZC3H12A, is a molecule known to be essential in controlling immune responses by regulating mRNA decay [66] and has been reported to destabilize IL6 mRNA [65]. ZC3H12A was upregulated by BRO. Thus, BRO seems to support degradation of IL6 mRNA, which could be causing the strong suppression of protein levels, despite unaffected transcription. Importantly, in the last decade, the role of IL6 trans-signaling via soluble IL6 receptor emerged as prominent regulator of inflammation [67,68]. Basically, the classical pathway has been associated with anti-inflammatory functions of IL6, as only few cells express both IL6 receptor (IL6R) and gp130 (IL6ST), which are both required to respond to IL6. Most cells express only gp130 and therefore are not responsive to IL6 alone. However, if IL6 is bound to soluble IL6 receptor, which is generated by cleavage via ADAM metallopeptidase domain 17 (ADAM17) during inflammatory processes, it can bind to gp130 and mediate the so-called trans-signaling. This mode of activation has been associated with pro-inflammatory functions of IL6 [67,69]. However, the trans-signaling of IL6 seems much more complex and further studies are needed to fully understand its regulation [68]. Interestingly, we observed in mouse PCLS no induction of *Il6r* or *Il6st* gene expression upon virus infection, but BRO upregulated both *Il6r* and *Il6st*, as well as *Adam17* in the presence but also in the absence of virus. This suggests that BRO supports IL6 trans-signaling, which would be contrary to the overall observed anti-inflammatory effects. However, IL6/sIL6R has been described to stimulate endothelial cell recruitment of mononuclear phagocytic cells involved in non-phlogistic removal of apoptotic neutrophils and resolution of the inflammation [67,70]. Thus, in line with the observed stimulation of molecules involved in recruitment and activation of leukocytes, BRO could potentially exhibit its beneficial effects also via stimulating phagocyte infiltration via IL6 trans-signaling.

In summary, our data obtained from mouse PCLS infection ex vivo suggest that BRO exhibits its beneficial effects in treating inflammatory respiratory diseases such as the rhinovirus induced common cold, by differentially modulating and supporting the host immune response while limiting excess inflammation. Future studies in human PCLS are needed to verify whether our findings on BRO’s mode-of-action in the early events of infection translate to the human situation. More important is also to investigate the therapeutic effect of BRO when applied after the onset of infection. Furthermore, as BRO exerted strong modulatory effects independent of RV infection, its mode-of-action presumably can be beneficial to balance the immune response to other respiratory viruses.

## 4. Materials and Methods

### 4.1. Media, Chemicals, and Reagents

Dulbecco’s phosphate buffered salt solution (DPBS) was obtained from Lonza (Wuppertal, Germany). Dulbecco’s minimal essential medium (DMEM), supplemented with 100 U/mL penicillin and streptomycin, all obtained from Gibco (Life Technologies, Darmstadt, Germany) was used as medium for PCLS preparation. For infection experiments, the following medium—further referred to as ‘culture medium’ was used: Gibco Minimal Essential Medium (1× MEM, Life Technologies, Darmstadt, Germany) was supplemented with 2% penicillin/streptomycin (Sigma Aldrich, Munich, Germany), 2 mM l-glutamine and non-essential amino acids (1× NEAA; both Gibco, Life Technologies, Darmstadt, Germany). Low-gelling temperature agarose, protease inhibitor cocktail P1860, Triton X-100, and Earle’s Balanced Salt Solution (EBSS) were purchased from Sigma-Aldrich (Munich, Germany). Human rhinovirus 1b was obtained from Virapur (Lot J1323A, San Diego, CA, USA). The LDH Cytotoxicity Assay Kit was obtained from Roche (Mannheim, Germany). The Pierce™ BCA (bicinchoninic acid) total protein kit was purchased from Thermo Scientific/Life Technologies (Darmstadt, Germany).

### 4.2. Test Item

The test item Bronchobini^®^’s ingredient solution (BRO) was manufactured and provided by Heel GmbH (Baden-Baden, Germany). Bronchobini^®^, a low dose multi-component multi-target medication, contains five components in different dilutions: *Atropa belladonna* (D5), *Bryonia* sp.(D3), *Cetraria islandica* (D3), *Drosera* (D3), *Psychotria ipecacuanha* (D3), and ethanol concentration 9.2% (m/m). Bronchobini^®^’s active substances are very low concentrated. Therefore, pharmacological studies have not been conducted and are not required for regulatory or legal reasons. To cover an appropriate dosage range to investigate the effects of BRO, the test item was diluted in an incubation medium to a final dilution of 1:10 (high dose), 1:100 (medium dose), and 1:1000 (low dose), respectively. Vehicle (9.2% ethanol in purified water) was used at a final dilution of 1:10 in culture medium (final ethanol concentration 0.92%). Working solutions were freshly prepared immediately before usage.

### 4.3. Animals

Female Balb/c mice, (6–8 weeks) were obtained from Charles River (Sulzfeld, Germany). Animals were housed under conventional housing conditions (22 °C, 55% humidity, and 12 h day/night rhythm). Diet (commercial pellets: Ssniff R/M-H V1534, Ssniff-Spezialdiäten, Soest, Germany) and drinking water were available ad libitum. Animal health was checked daily. Animals were acclimated for at least 2 weeks. For removal of lungs for preparation of PCLS, animals (age 8-10 weeks) were sacrificed by anaesthesia with an i.p. overdose (160mg/kg, 1 ml/kg) of pentobarbital sodium (Narcoren®, Merial GmbH, Hallbergmoos, Germany) and exsanguination via the vena cava caudalis. Sacrifice of the animals for organ removal was registered at the responsible authority (Lower Saxony Federal State Office for Consumer Protection and Food Safety), and performed in accordance with the Regulations of the German Animal Protection Law (Tierschutzgesetz of 18 May 2006, BGBl. I S. 1206, 1313; adopted 28 July 2014, BGBl.I S. 1308) and European Council Directive on the protection of animals used for scientific purposes (2010/63/EU). 

### 4.4. Preparation of Precision-Cut Lung Slices (PCLS)

Precision-cut lung slices (PCLS) were prepared as described previously [46,47,50,71]. Briefly, lungs were inflated using agarose/medium solution and cooled on ice. After polymerization of the agarose, slices of 350 µm were cut and collected in 4 °C cold EBSS, using an automatic oscillating tissue slicer (OTS 5000, Warner Instruments, Hamden, CT, USA). Tissue slices were transferred to petri dishes filled with DMEM+P/S and incubated under standard cell culture conditions (37 °C, and 5 % CO_2_). The medium was exchanged at least four times every 30 min for 2–3 h to remove cell debris. Only PCLS containing an airway with an intact smooth muscle layer and active beating of the cilia were selected for the study. As the number of airway-containing mPCLS that can be generated from one donor mouse is limited, for each experiment PCLS from three mice were pooled.

### 4.5. Virus

The obtained human rhinovirus (RV) serotype 1b batch was aliquoted and frozen at −80 °C. The working solution of the frozen virus stock was prepared freshly before use in the culture medium to 2 × 10^5^ IU/mL. UV-inactivated RV was used as a replication-deficient negative control.

### 4.6. Rhinovirus Infection and Pharmacological Treatment of PCLS

Infection experiments were performed at 33 °C as optimal replication temperature for RV. PCLS were pre-incubated with BRO in three different doses (diluted 1:10, 1:100, and 1:1000 in culture medium) or BRO vehicle for 1 h at 33 °C, 5 % CO_2_. Afterwards, the medium was removed, fresh treatment solutions added (250 µL per well), and PCLS were infected with 250 µL 2 × 10^5^ IU/mL RV, UV-inactivated RV as replication-deficient negative control or culture medium as uninfected control. Duplicate wells with two PCLS each were used per condition and endpoint. PCLS were incubated at 33 °C, 5% CO_2_ for 24 h. After 24 h supernatants and tissue lysates or snap-frozen PCLS were collected for analysis of tissue viability, virus load, cytokine release, and gene expression analysis.

### 4.7. Analysis of Tissue Viability

Tissue viability was determined using an LDH Cytotoxicity Assay Kit according to the manufacturer’s instructions. Briefly, LDH was detected in supernatants of tissue cultures using 50 µL of supernatant incubated with 50 µL of reagent mix for 20 min at RT in the dark. Absorption was detected with 492 nm and a reference wavelength of 630 nm.

### 4.8. Total Protein Determination

PCLS were lysed using 1% Triton-X100 in DPBS supplemented with 0.2% proteinase inhibitor cocktail for 1 h at 4 °C. Total protein content of the PCLS tissue lysates (not including the supernatant) was determined using the Pierce BCA protein assay kit (Thermo Scientific, Rockford, IL, USA) according to the manufacturer’s instructions as described previously [48].

### 4.9. Determination of Cytokines

For the measurement of cytokines, supernatants were collected from tissue cultures and supplemented with 0.2% protease inhibitor cocktail. Mouse IFN-gamma (IFNG), IL1B, IL6, KC/GRO, IL10, and TNF were analyzed using MSD multiplex and CCL5 using MSD single plex tissue culture kits (Mesoscale Discovery, Gaithersburg, MD, USA). Mouse CXCL10, CCL2, IFNA, and IFNB were measured using commercially available ELISA (R&D Systems, Wiesbaden, Germany). The analysis was performed in duplicates for each sample according to the manufacturer’s instruction. The MSD assays were performed using an MSD Sector Imager 2400. The calculation of cytokine concentration was based on a four-fold serial diluted standard. Data analysis was conducted using the Discovery Workbench software (version 4.0, Mesoscale Discovery, Gaithersburg, MD, USA). Detection limits of the assays are given in Table S7. The cytokine content of each sample was related to the total protein content of the PCLS (pg cytokine/ mg total protein).

### 4.10. Analysis of Virus Load

The determination of virus load in PCLS homogenates was performed by dilution plating on HeLa cells and determination of the tissue culture infective dose 50 (TCID_50_). Therefore, PCLS were disintegrated with 1 mL ice cold PBS using FastPrep^®^ lysing matrix D tubes (MP Biomedicals, Santa Ana, CA, USA). The tissue homogenate suspension was centrifuged for 5 min at 500× *g* and the supernatant was frozen at −80 °C until determination of virus load by dilution plating on HeLa cells. After incubation at 33 °C, 5 % CO_2_ for 72 h and staining with 0.1% crystal violet, the cytopathic effect was assessed and TCID_50_ was calculated as previously described [49].

The qPCR experiments were performed using the TaqMan-based HRV1B genesig^®^ standard real-time PCR detection kit (Primerdesign™ Ltd., Eastleigh, UK) and the oasig™ One Step qRT-PCR MasterMix (Primerdesign™ Ltd., Eastleigh, UK ) according to the manufacturer’s instructions. The following one step amplification protocol was used: Reverse transcription 10 min 55 °C, enzyme activation 2 min 95 °C, denaturation 10 s 95 °C + data collection 60 s 60 °C for 50 cycles. Data collection was performed through the FAM channel. To enable quantitative analysis a standard curve was recorded started with 2 × 10^5^ copies/µL with 1:10 dilutions until 2 copies/µL. 10 ng RNA was analyzed in a final reaction volume of 20 µL. Resulting Ct-values were converted to copy numbers per reaction using the standard curve.

### 4.11. RNA Isolation and Quality Analysis

RNA was isolated according to an optimized protocol for RNA isolation from PCLS from different species [72]. The protocol is based on a specific homogenization and phenol extraction procedure coupled with a MagMax™ magnetic beads (ThermoFisher Scientific, Dreieich, Germany) cleaning procedure. RNA concentration (A260) and purity (A260/A280 ratio) were measured by spectrophotometry (NanoDrop™ 2000 Spectrophotometer, software version 1.6.198, ThermoFisher Scientific, Dreieich, Germany). RNA integrity number (RIN) was evaluated using an Agilent 2100 Bioanalyzer^®^ (Agilent Technologies, Ratingen, Germany). All RNA samples showed good quality as indicated by high RIN values between 7.5 and 10.0.

### 4.12. Transcriptome Arrays

Transcriptome analyses were done using the Affymetrix GeneChip™ Whole Transcript (WT) PLUS Reagent Kit and the GeneChip™ Mouse Transcriptome Arrays 1.0 (also named Clariom™ D Array) according to the manufacturer’s recommendation (ThermoFisher, Dreieich, Germany). A total RNA of 100 ng was used as a starting material for target preparation. Arrays were subsequently washed, stained, and scanned using the Affymetrix GeneChip™ Command Console Software (ThermoFisher) with .cel files as data output. Raw data were deposited at GEO database (GSE126832).

Data analysis was carried out using Transcriptome Analysis Console (TAC) Software 4.0 (ThermoFisher). Specifically, normalization, probe summarization, and data quality control of the MTA-1.0 microarrays were undertaken with TAC 4.0 according to manufacturer’s specifications. Microarray data were normalized by the RMA method, and subjected to subsequent analysis and visualization of gene level differential expression. Based on the results of the whole-genome transcriptome arrays and determination of differentially expressed genes (DEGs), further bioinformatic analyses were performed using Ingenuity Pathway Analysis Software (IPA, Qiagen, Venlo, The Netherlands).

### 4.13. Statistics

Data in the bar graphs are given as means + SD for *n* = 3 experiments, with individual plots representing the mean of biological duplicates within each experiment. For analysis of significant differences between multiple groups ANOVA with paired matching as same batch of PCLS was used within one experiment) with Sidak’s post test was used (Software: GraphPad Prism, version 8.0.1). Differences between groups were considered statistically significant at the level of *p* ≤ 0.05. Statistics for gene expression and pathway analysis was performed as describe above.

## Figures and Tables

**Table 1:** Summary of differentially expressed genes (DEGs) obtained after genome-wide transcriptome analysis of ex vivo RV-infected mPCLS.

Comparison		Total DEGs *	Up Regulated	Down Regulated
**BRO effect under baseline condition**				
(Medium/Vehicle)	(Medium/Medium)	37	14	23
(Medium/BRO 1:10)	(Medium/Vehicle)	6693	2497	4196
**RV induced gene regulation**				
(RV/Vehicle)	(RV/Medium)	1	1	0
(RV/Vehicle)	(Medium/Vehicle)	692	631	61
**BRO effect on RV infection**				
(RV/BRO 1:10)	(RV/Vehicle)	5665	2220	3445
(RV/BRO 1:100)	(RV/Vehicle)	1257	446	811
(RV/BRO 1:1000)	(RV/Vehicle)	71	9	62

* Differentially-expressed genes (DEGs) were obtained with the criteria fold change <−2 or fold change >2; and ANOVA *p*-value < 0.05, based on list of genes by using Transcriptome Analysis Console Software (TAC 4.0). Raw data are deposited at GEO database (GSE126832).

**Table 2:** BRO modulation of NFkB target genes and related molecules and their biological functions.

Gene	Fold Change		Gene	Fold Change	
	RV vs. Med	RV+BRO vs. RV		RV vs. Med	RV+BRO vs. RV
**Proinflammatory chemokines/cytokines**			**Leukocyte activation**		
*Ccl2*	5.08 *	−6.20 *	*Cd40*	7.77 *	1.40
*Ccl5*	34.23 *	−1.50	*Cd69*	5.99 *	−1.52
*Cxcl10*	178.71 *	−6.27 *	*Icos/Icosl*	1.03/2.43	2.77 */5.90 *
*Il1b*	1.61	−2.49 *	**Apoptosis**		
*Casp1*	2.99 *	−2.83 *	*Tnfsf10*	14.66 *	−61.82 *
*Il6*	15.56 *	−1.68	*Tnfaip3*	3.71 *	23.7 *
*Il6r*	−1.19	4.30 *	*Casp8*	1.43	−2.14 *
*Il6st(gp130)*	−1.34	2.98 *	*Traf1/2*	1.67/1.38	6.4 */6.5 *
*Adam17*	−1.10	2.43 *	**Inducible enzymes and prostaglandin**		
*Tnf*	4.05 *	2.60 *	*Nos2*	3.76 *	−28.68 *
**Leukocyte infiltration**			*Ptgs2*	2.88 *	2.94 *
*Icam1*	2.25 *	7.66 *	*Ptges*	1.13	−4.40 *
*Vcam1*	8.36 *	2.16 *	*Namp*	5.03 *	−3.02 *

* Fold change <−2 or fold change >2; and ANOVA *p*-value < 0.05.

## Supplementary Materials

Supplementary materials can be found at https://www.mdpi.com/1422-0067/20/9/2242/s1.

## Abbreviations

BRO	Bronchobini^®^’s ingredients
COPD	Chronic obstructive pulmonary disease
DEG	Differential expressed genes
DMEM	Dulbecco’s modified Eagle’s medium
DPBS	Dulbecco’s phosphate buffered salt solution
EBSS	Earle’s balanced salt solution
FAM	Carboxyfluorescein
FC	Fold change
IPA	Ingenuity pathway analysis software
IU	Infectious units
LDH	Lactate dehydrogenase
LDLR	Low density lipoprotein receptor
LPS	Lipopolysaccharides
MEM	Minimal essential medium
mPCLS	Mouse precision-cut lung slices
MSD	Mesoscale Discovery
MTA	Mouse transcriptome arrays
NEAA	Non-essential amino acids
PCA	Principal Component Analysis
qPCR	Quantitative polymerase chain reaction
RIN	RNA integrity number
RV	Rhinovirus
TAC	Transcriptome analysis console software
TCID_50_	Tissue culture infective dose 50
