# Supplementary material for: Transcriptomic Analysis Reveals Priming of The Host Antiviral Interferon Signaling Pathway by Bronchobini® Resulting in Balanced Immune Response to Rhinovirus Infection in Mouse Lung Tissue Slices
Source: Int J Mol Sci. 2019 May 7;20(9):2242. doi: 10.3390/ijms20092242 (PMC6540047; doi:10.3390/ijms20092242)
Supplement: Supplementary file 1 [file ijms-20-02242-s001.zip › Supplement_Reamon-Buettner et al_Bronchobini modulation of RV infection.docx]

Supplementary Materials IJMS_ Reamon-Buettner et al.

**BRO did not impair tissue viability**

Upon treatment with different doses of BRO or vehicle and infection with RV, UV-RV or sham-infection, the tissue viability was maintained in all slices during the culture up to 24h p.i. as indicated by the low LDH release of approx. 20-35% compared to the maximum LDH release of the TritonX-100 lysed control set to 100% (Figure S1). In RV-infected slices the LDH level was slightly higher, which is a known effect that presumably is a result of RV preparation from lysed infected cells releasing LDH.

**
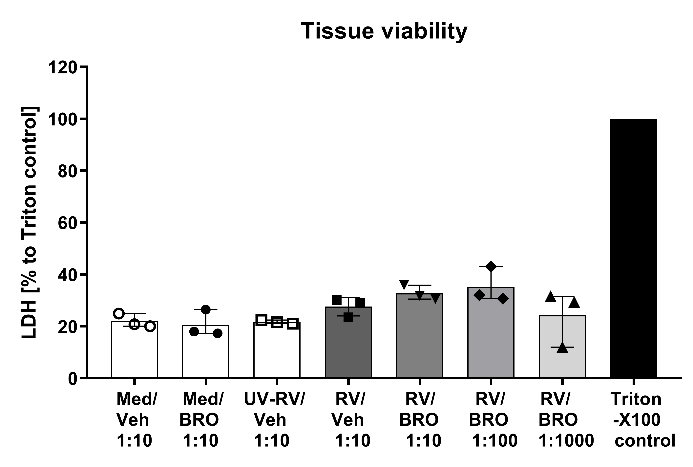
**

**Figure S1:** BRO did not impair tissue viability. LDH release is given as % to Triton-lysed control (set to 100%). Scatter plots with bars show mean + SD for n=3 independent experiments with individual plots showing mean of two biological replicates (duplicate wells with 2 PCLS each) per experiment. Each experiment was performed with PCLS pooled from three mice.

**BRO treatment did not alter virus load**

The viral load was quantified as infectious units (IU) assessed by dilution plating on HeLa cells and calculated based on tissue culture infective dose 50 (TCID_50_) according to the observed cytopathic effects. In all RV-infected samples, infectious viral particles were recovered, but with a low titer (approximately 1x10^2), while non-infected or sham-infected (medium and UV-RV infected) controls were negative. The amount of virus was not significantly altered by treatment with BRO in all concentrations (Figure S2).


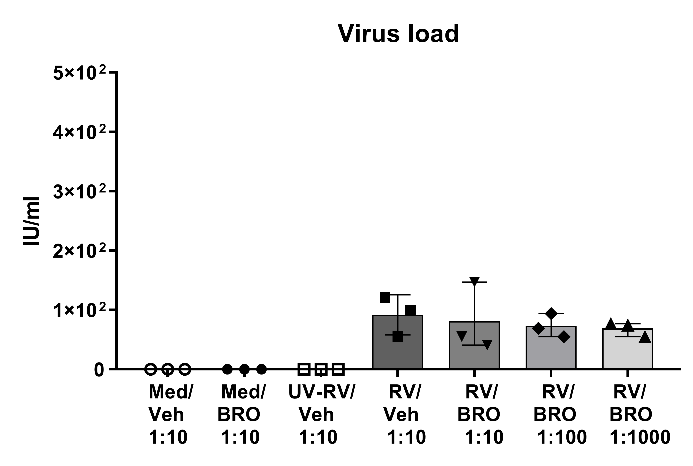


**Figure S2:** BRO treatment did not alter infectious virus titer. Virus load was determined by dilution plating of PCLS tissue homogenate on HeLa cells and the tissue culture infective dose 50 (TCID_50_) was calculated. Scatter plots with bars show mean + SD for n=3 independent experiments with individual plots showing mean of two biological replicates (duplicate wells with 2 PCLS each) per experiment. Each experiment was performed with PCLS pooled from three mice.

As only low viral titers could be detected with the TCID50 assay, we additionally performed PCR quantification of virus of the infected samples as a more sensitive method. Virus quantification by PCR confirmed that BRO did not alter the virus load (Figure S3).


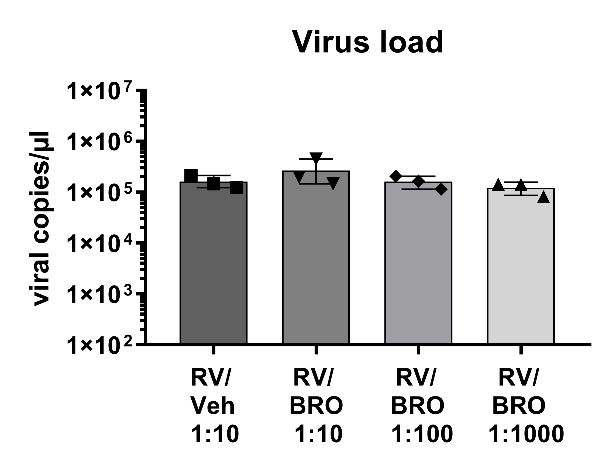


**Figure S3:** BRO treatment did not alter total virus load. Virus load was determined by TaqMan-based RV1b real-time PCR. Scatter plots with bars show mean + SD for n=3 independent experiments with individual plots showing mean of two biological replicates (duplicate wells with 2 PCLS each) per experiment. Each experiment was performed with PCLS pooled from three mice.

**BRO modulation of gene expression**

**
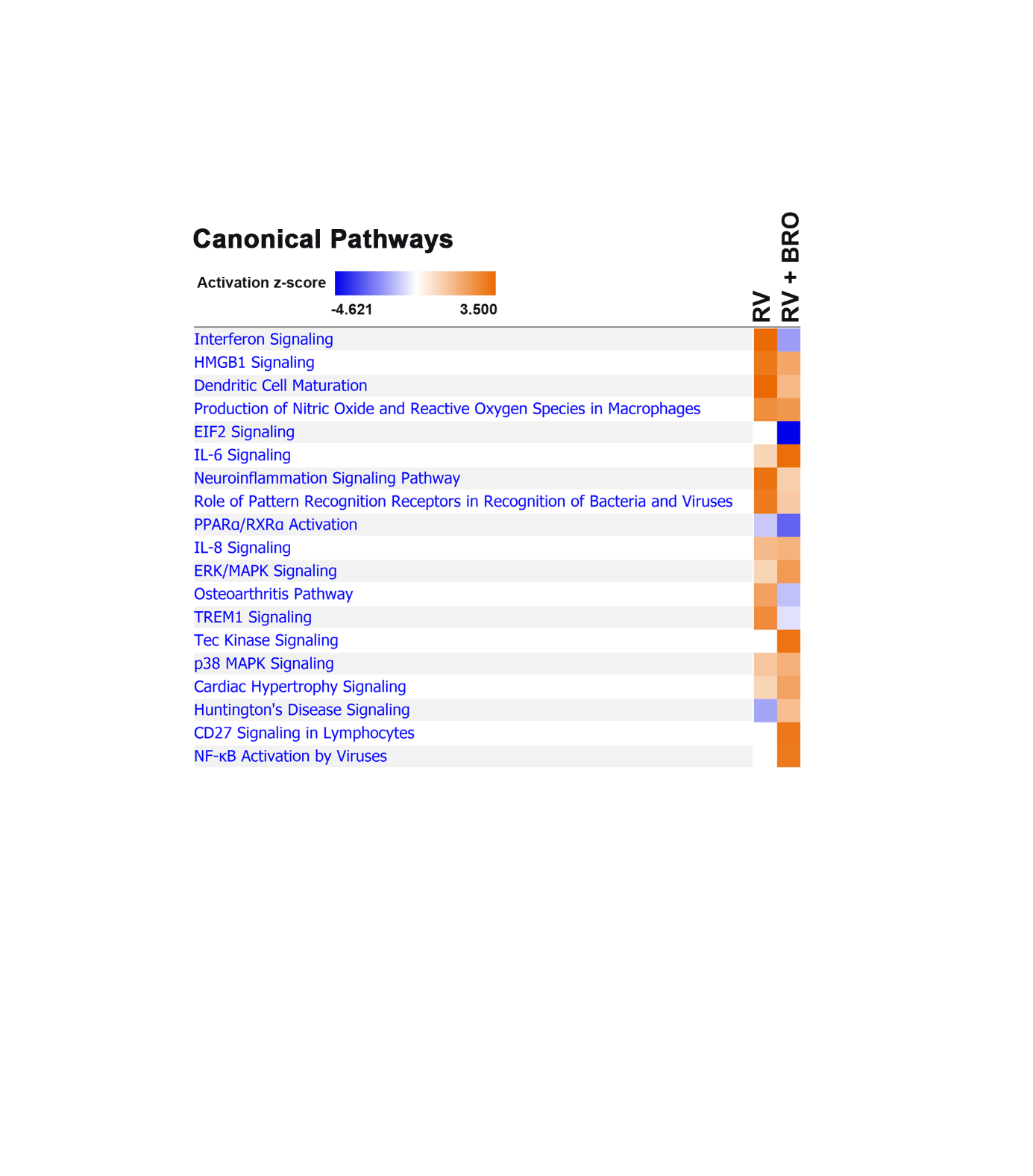
**

**Figure S4.** Heat map of top 10 regulated canonical pathways. Comparison analysis of canonical pathways regulated by RV infection and BRO treatment, was performed with Ingenuity Pathway Analysis. Activation (orange); Inhibition (blue).


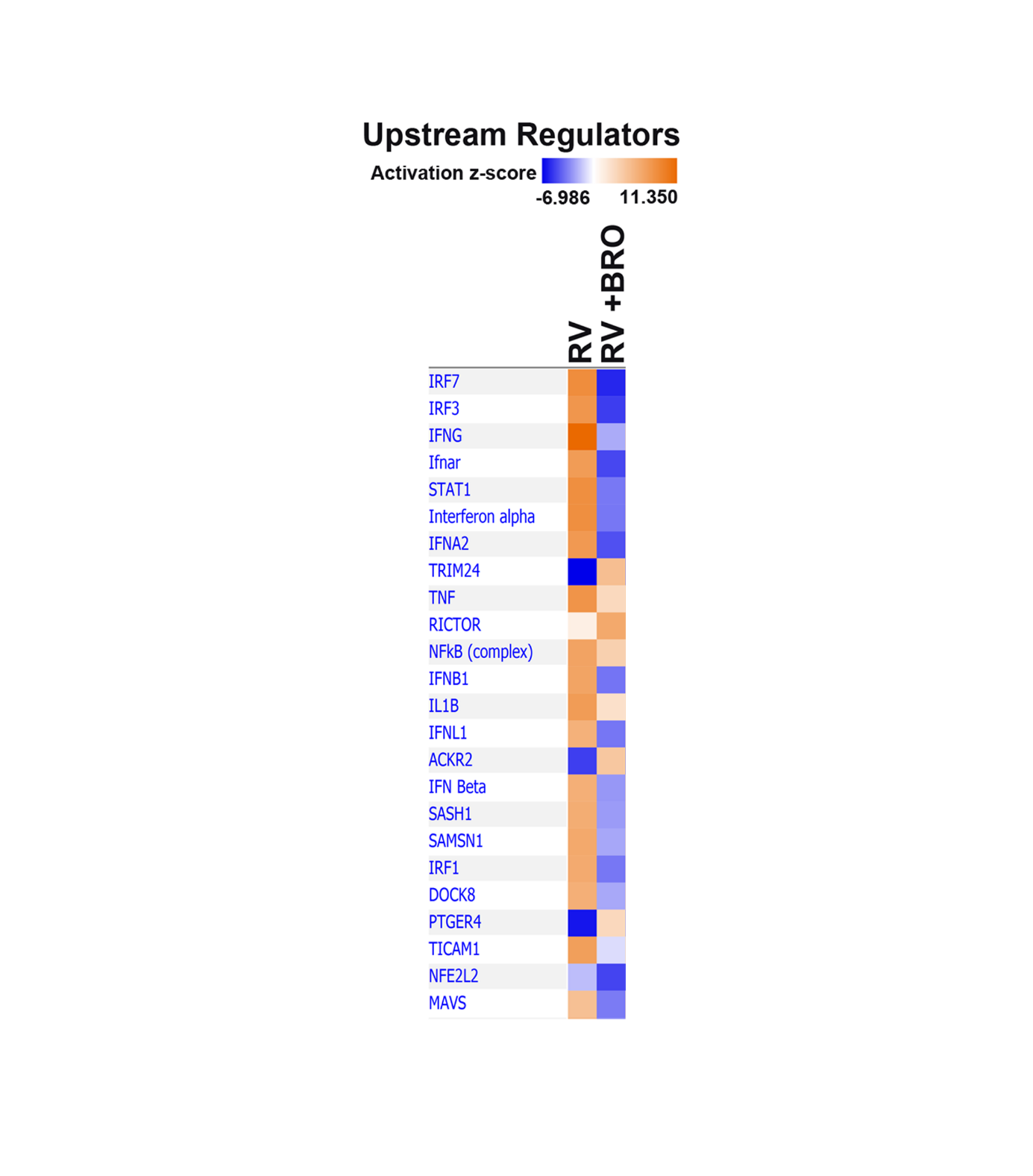


**Figure S5.** BRO modulates expression of upstream regulators of RV-induced interferon signaling pathway. Heatmap and predicted upstream regulators of the IFN signaling pathway, obtained using Ingenuity Pathway Analysis. Orange (activation), blue (inhibition).

**
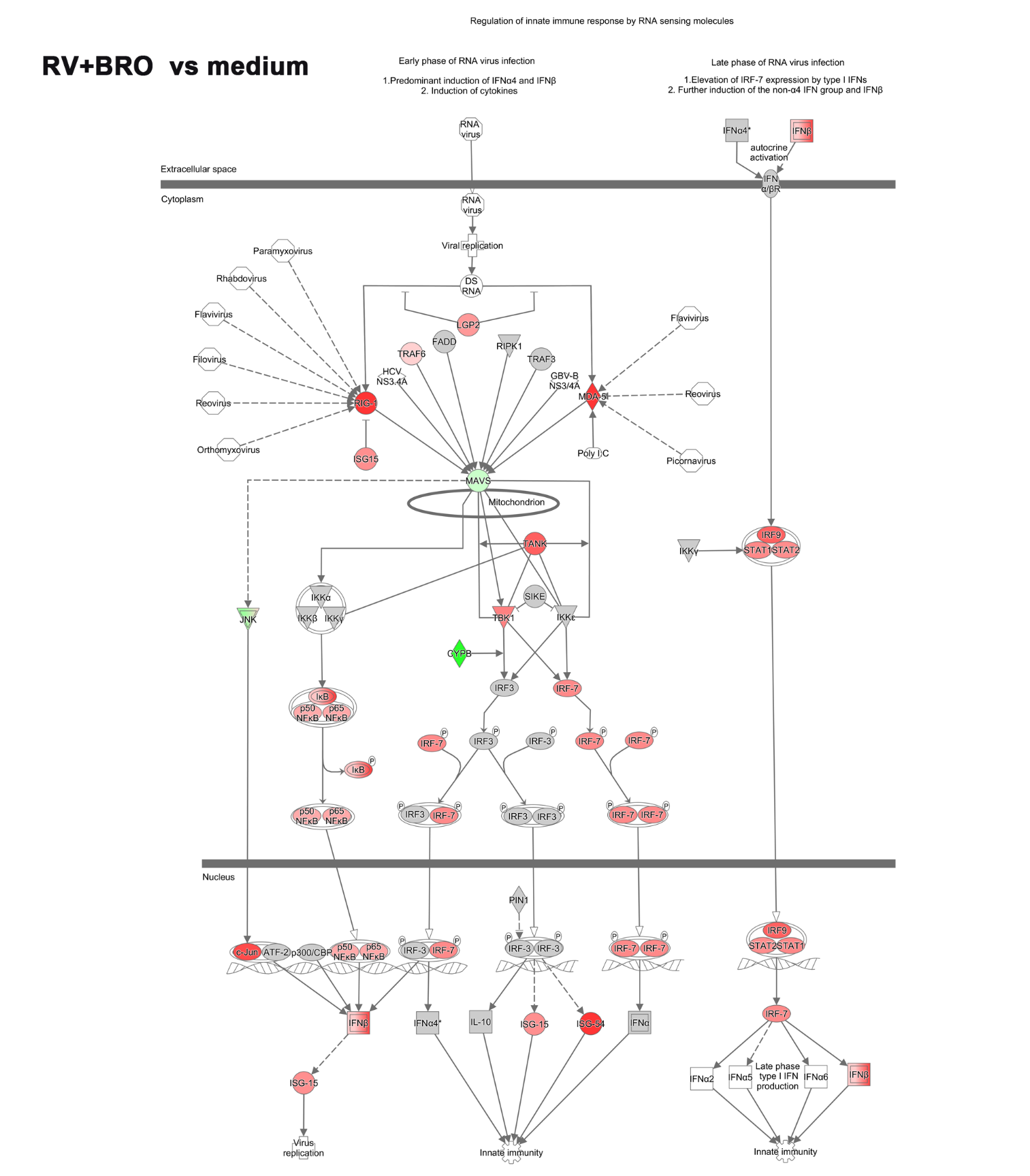
**

**Figure S6.** BRO reduces but does not completely shut down the RV-induced interferon response activating pathway. Regulation of interferon response factor (IRF) activation by PRR (pattern recognition receptors) in RV+BRO versus uninfected medium control. Pathway obtained using Ingenuity Pathway Analysis. Red (upregulated), green (downregulated, gray or white (does not meet cut-off criteria or not involved in the pathway).

**Supplement Tables**

The following supplement tables are provided as excel files:

Table S1: Common DEGs after comparison of condition RV with RV+BRO

Table S2: RV unique DEGs after comparison of condition RV with RV+BRO

Table S3: RV+BRO unique DEGs after comparison of condition RV with RV+BRO

Table S4: DEGs of IRF PRR pathway comparison RV, RV + BRO, Med + Vehicle, Med + BRO

Table S5: DEGs activation of IRF pathway BRO vs Med (uninfected) and comparison

Table S6: DEGs activation of NFkB pathway BRO vs Med (uninfected) and comparison

**Table S7:** Detection limits of MSD (IFNG, IL1B, IL6, KC, IL10, TNF, CCL5) and ELISA (IFNA, IFNB, CCL2, CXCL10) are given in pg/ml for LLOD = lower limit of detection and ULOD = upper limit of detection.

|  | IFNG | IL1B | IL6 | KC | IL10 | TNF | CCL5 | IFNA | IFNB | CCL2 | CXCL10 |
| --- | --- | --- | --- | --- | --- | --- | --- | --- | --- | --- | --- |
| LLOD | 0.04 | 0.11 | 0.61 | 0.24 | 0.95 | 0.13 | 2.4 | 12.5 | 12.5 | 3.91 | 62.5 |
| ULOD | 938 | 1710 | 5860 | 1980 | 3180 | 674 | 10000 | 400 | 800 | 250 | 4000 |
