# Supplementary figures and images for: Transcriptomic Analysis Reveals Priming of The Host Antiviral Interferon Signaling Pathway by Bronchobini® Resulting in Balanced Immune Response to Rhinovirus Infection in Mouse Lung Tissue Slices
Source: Int J Mol Sci. 2019 May 7;20(9):2242. doi: 10.3390/ijms20092242 (PMC6540047; doi:10.3390/ijms20092242)

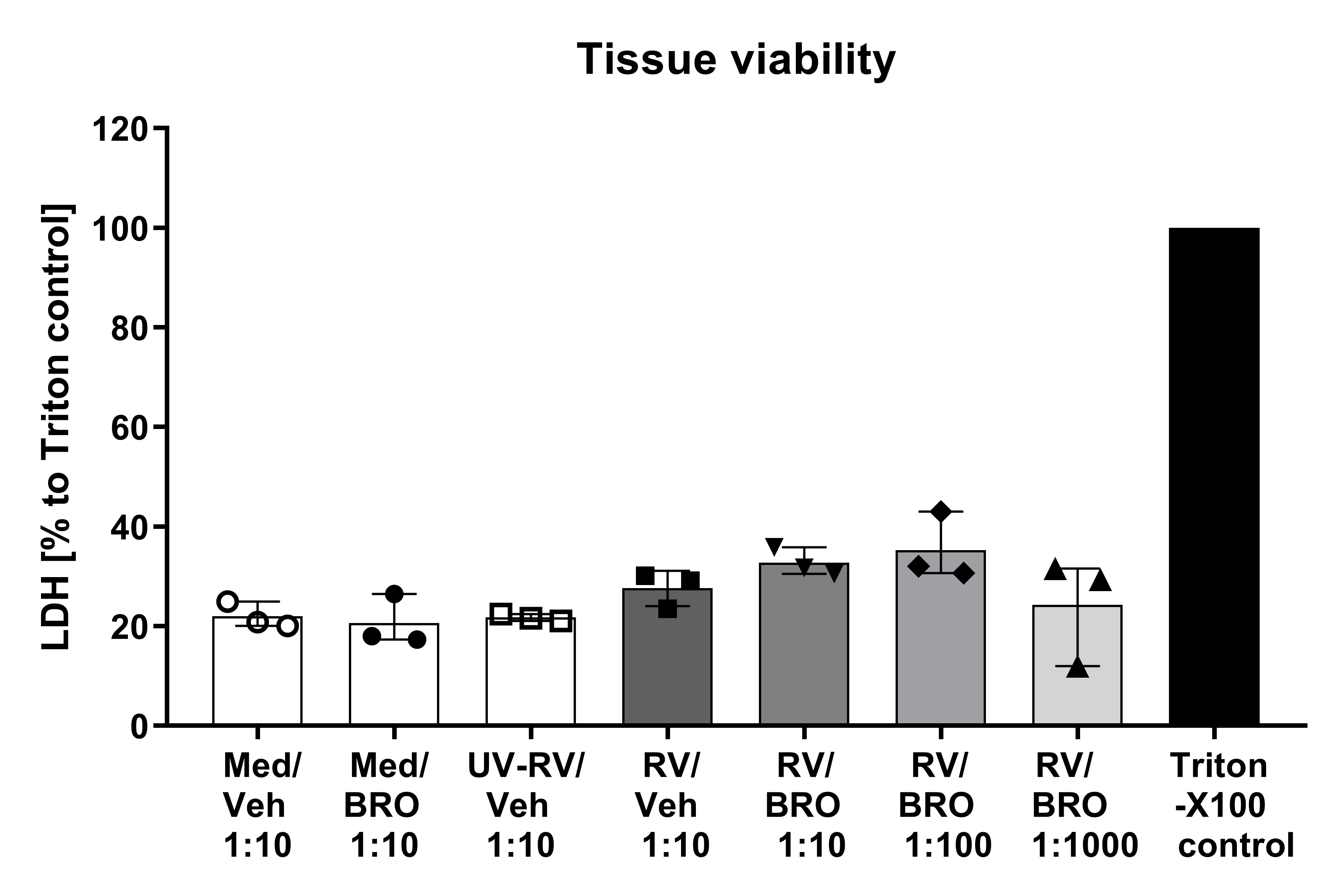

Supplement: Supplementary file 1 [file ijms-20-02242-s001.zip › FIGURE S1.tif]

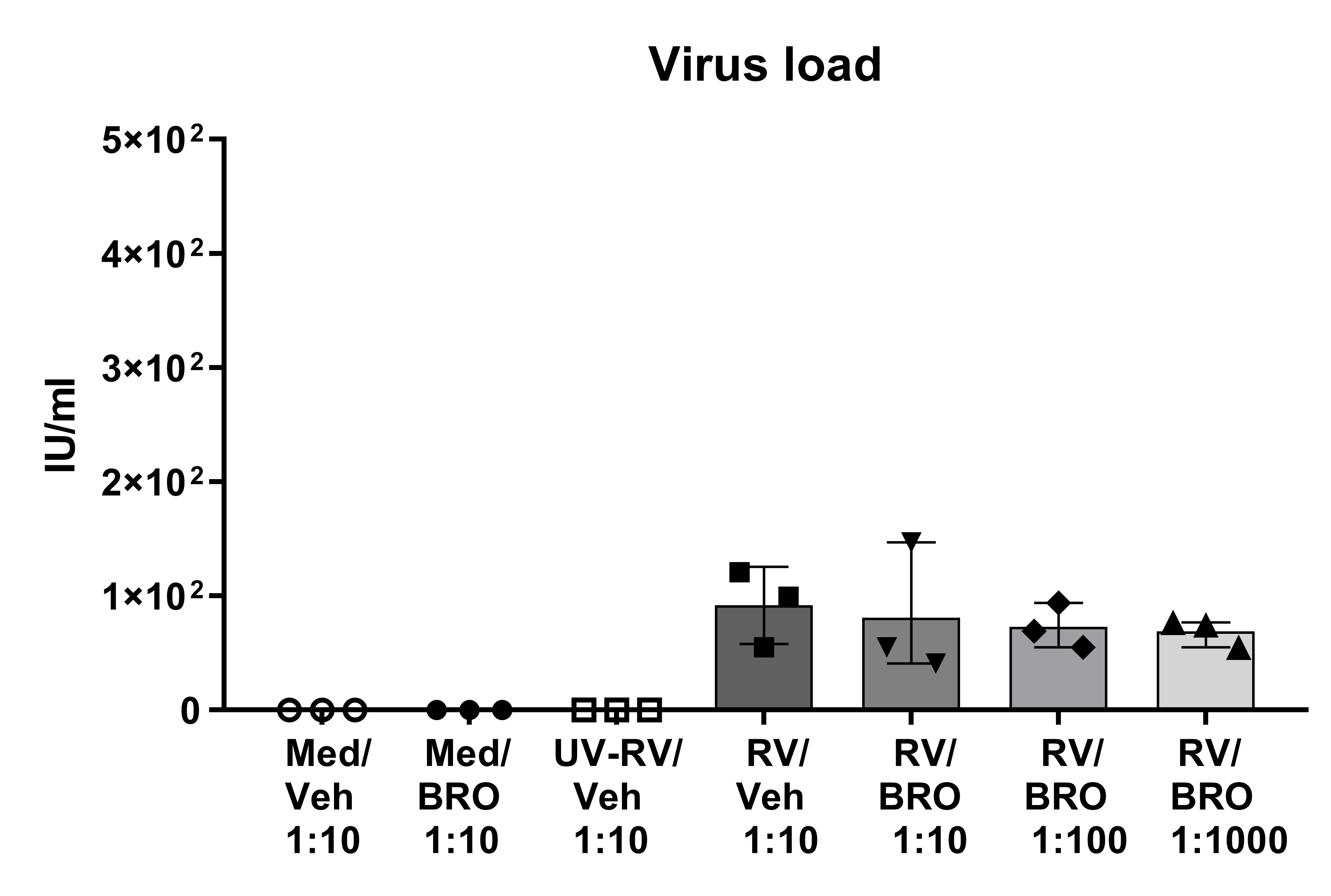

Supplement: Supplementary file 1 [file ijms-20-02242-s001.zip › FIGURE S2.tif]

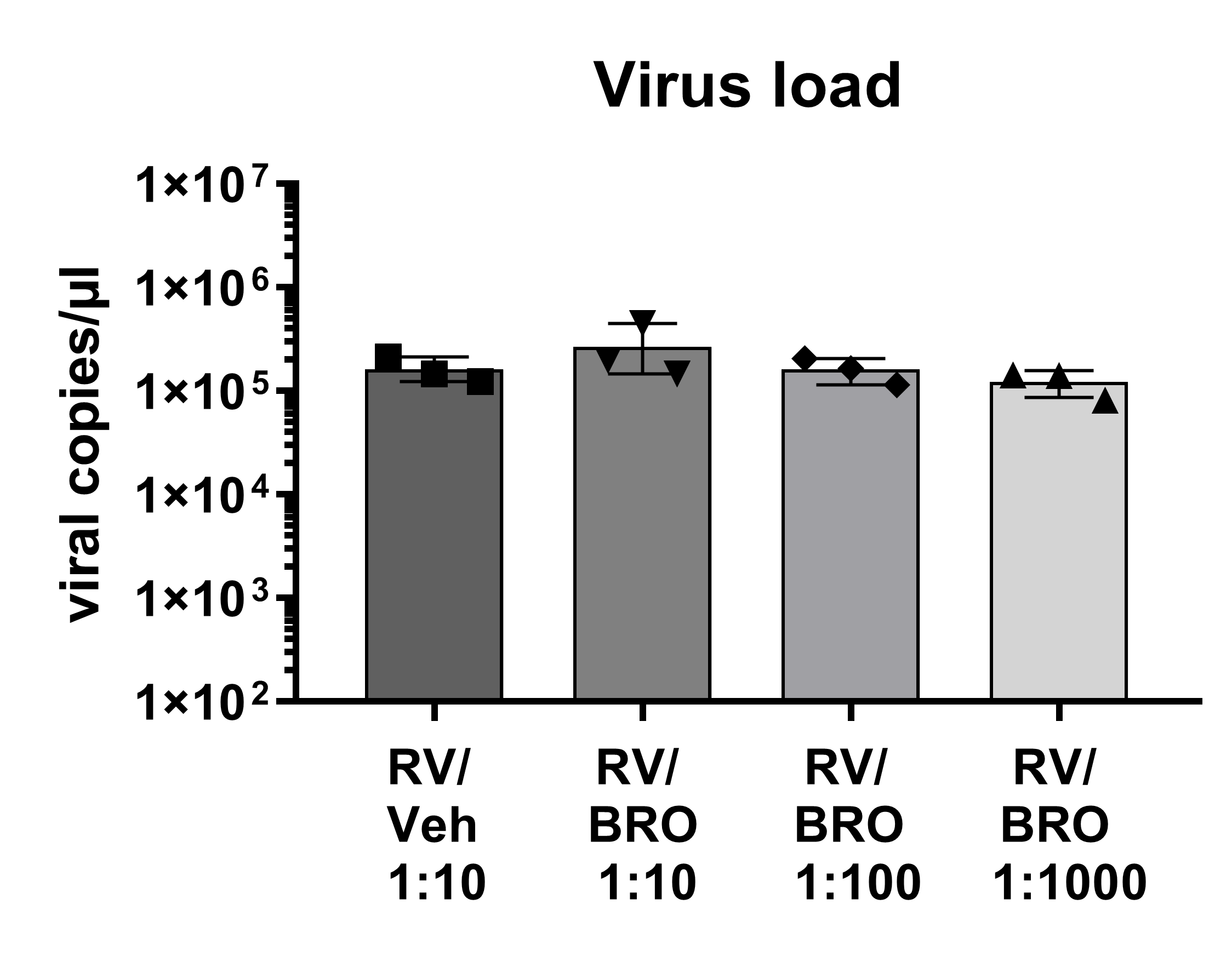

Supplement: Supplementary file 1 [file ijms-20-02242-s001.zip › FIGURE S3.tif]

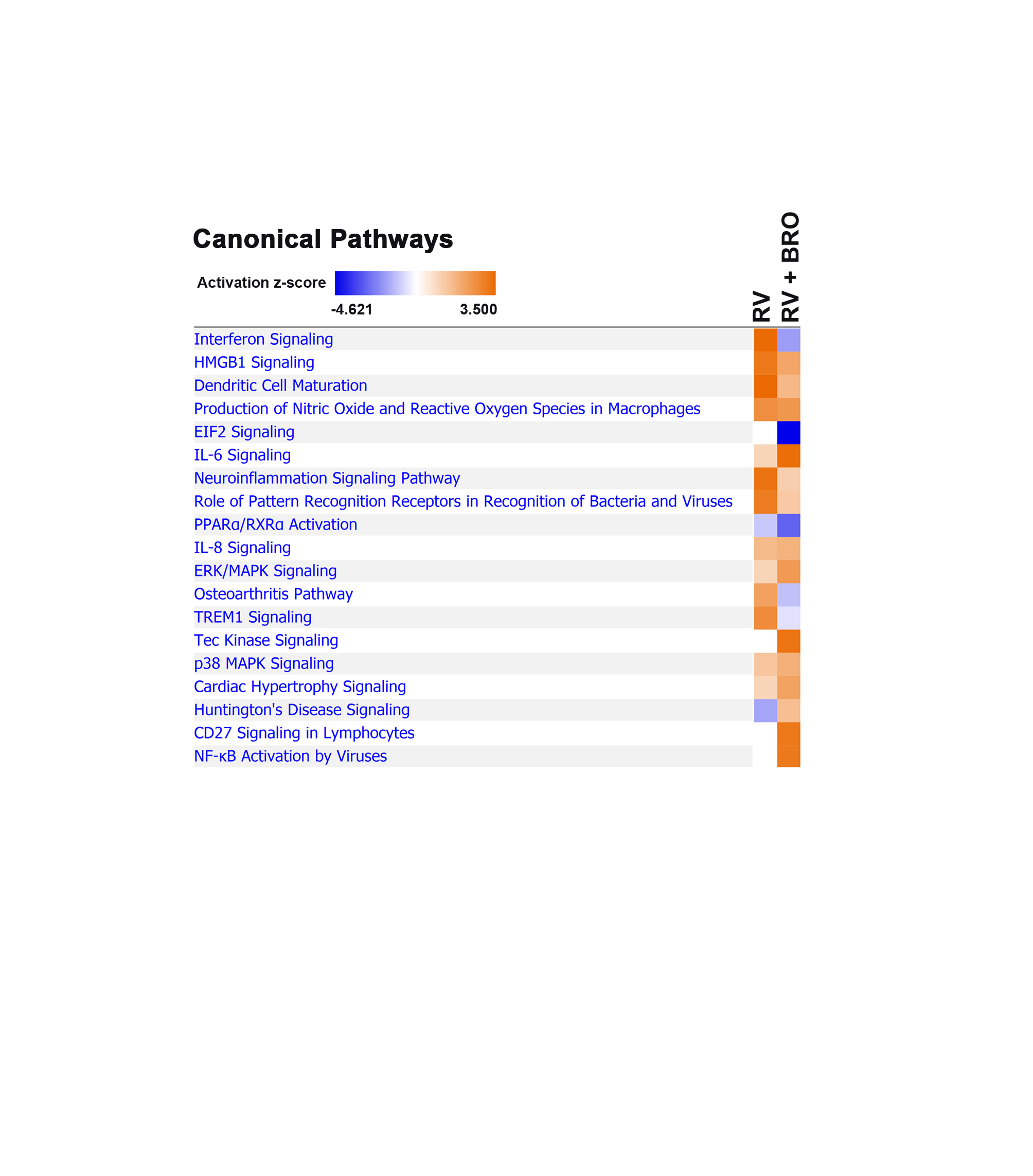

Supplement: Supplementary file 1 [file ijms-20-02242-s001.zip › FIGURE S4.tif]

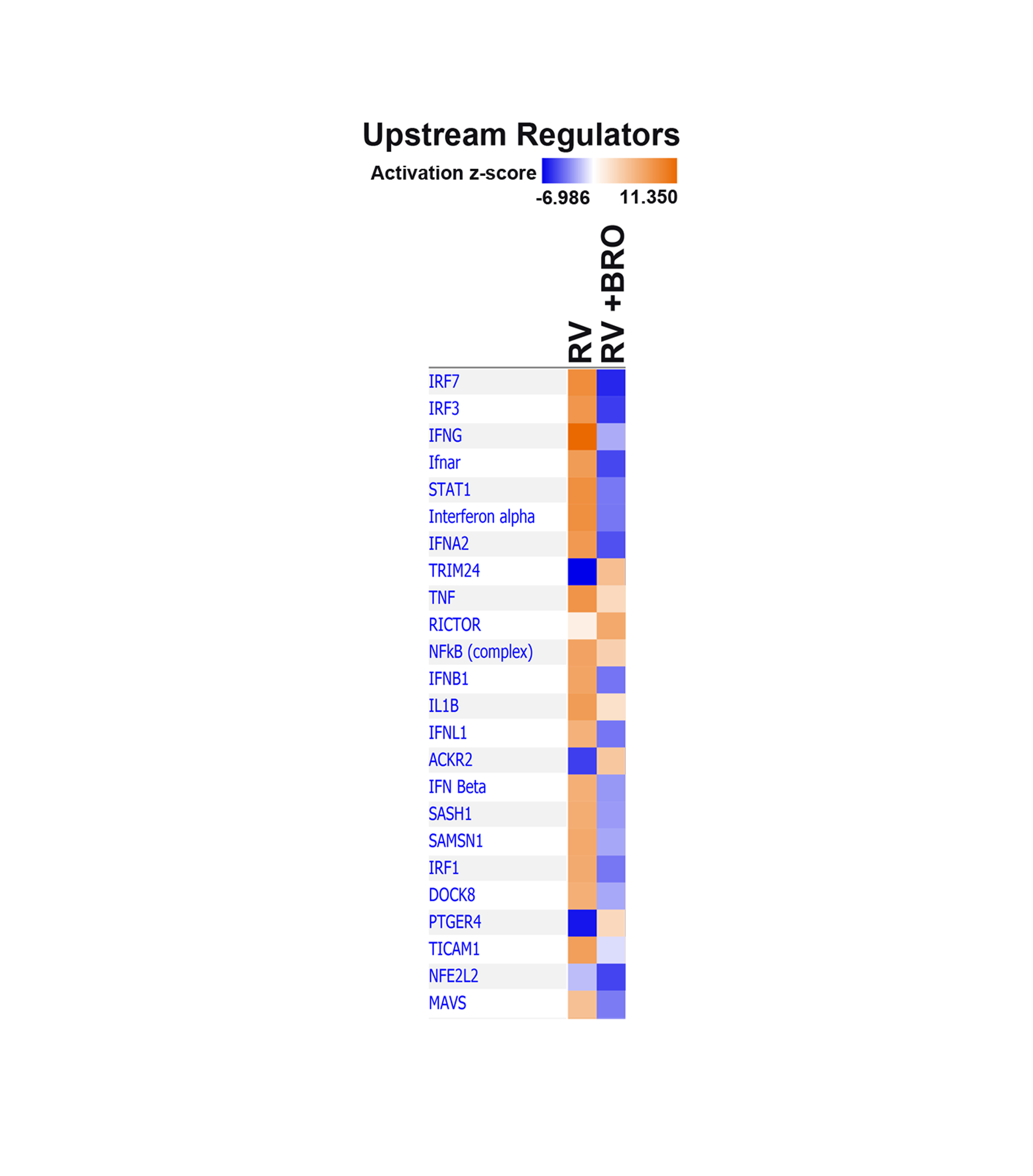

Supplement: Supplementary file 1 [file ijms-20-02242-s001.zip › FIGURE S5.tif]

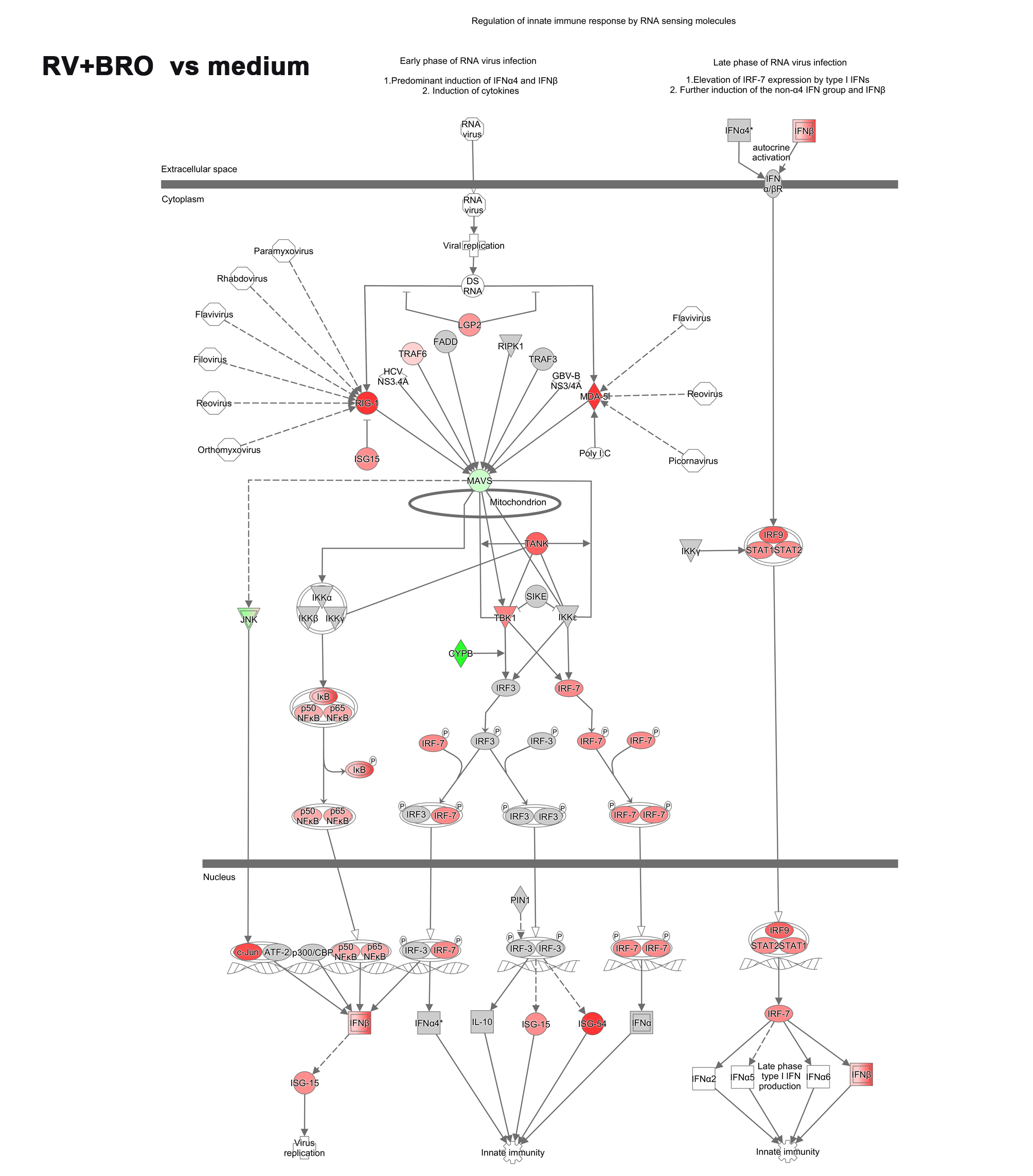

Supplement: Supplementary file 1 [file ijms-20-02242-s001.zip › FIGURE S6.tif]
